# Bridgehead effect and multiple introductions shape the global invasion history of a termite

**DOI:** 10.1038/s42003-021-01725-x

**Published:** 2021-02-12

**Authors:** Alexander J. Blumenfeld, Pierre-André Eyer, Claudia Husseneder, Jianchu Mo, Laura N. L. Johnson, Changlu Wang, J. Kenneth Grace, Thomas Chouvenc, Shichen Wang, Edward L. Vargo

**Affiliations:** 1 Department of Entomology, 2143 TAMU, Texas A&M University, College Station, TX USA; 2 Department of Entomology, Louisiana State University Agricultural Center, Baton Rouge, LA USA; 3 Ministry of Agriculture Key Lab of Molecular Biology of Crop Pathogens and Insect Pests, Institute of Insect Sciences, College of Agricultural and Biotechnology, Zhejiang University, Zhejiang, PR China; 4 Department of Veterinary Sciences, University of Wyoming, Laramie, WY USA; 5 Department of Entomology, Rutgers, The State University of New Jersey, New Brunswick, NJ USA; 6 Department of Plant and Environmental Protection Sciences, University of Hawaii at Manoa, Honolulu, HI USA; 7 Entomology and Nematology Department, Fort Lauderdale Research and Education Center, Institute of Food and Agricultural Sciences, University of Florida, Fort Lauderdale, FL USA; 8 Texas A&M AgriLife Genomics and Bioinformatics Service, College Station, TX USA

**Keywords:** Population genetics, Invasive species, Molecular ecology

## Abstract

Native to eastern Asia, the Formosan subterranean termite *Coptotermes formosanus* (Shiraki) is recognized as one of the 100 worst invasive pests in the world, with established populations in Japan, Hawaii and the southeastern United States. Despite its importance, the native source(s) of *C. formosanus* introductions and their invasive pathway out of Asia remain elusive. Using ~22,000 SNPs, we retraced the invasion history of this species through approximate Bayesian computation and assessed the consequences of the invasion on its genetic patterns and demography. We show a complex invasion history, where an initial introduction to Hawaii resulted from two distinct introduction events from eastern Asia and the Hong Kong region. The admixed Hawaiian population subsequently served as the source, through a bridgehead, for one introduction to the southeastern US. A separate introduction event from southcentral China subsequently occurred in Florida showing admixture with the first introduction. Overall, these findings further reinforce the pivotal role of bridgeheads in shaping species distributions in the Anthropocene and illustrate that the global distribution of *C. formosanus* has been shaped by multiple introductions out of China, which may have prevented and possibly reversed the loss of genetic diversity within its invasive range.

## Abstract (web summary)

Blumenfeld and Eyer et al. retrace the invasion history of the termite *Coptotermes formosansus* from eastern Asia to the US using approximate Bayesian computation. They find a complex invasion history, with multiple introductions originating from eastern Asia, as well as a bridgehead introduction originating from Hawaii, which appears to have mitigated the loss of genetic diversity within the invasive US range.

## Introduction

Biological invasions are a defining feature of the Anthropocene^1,2^, a byproduct of globalization where human transport and trade have facilitated the transfer of organisms throughout the world^3–5^. Remarkably, the accumulation of introduced species worldwide has yet to reach saturation^6^, and the harmful effects these invasive species have on the communities and ecosystems they invade cannot be overstated^7,8^. The success of invasive species in their new environments has often been considered paradoxical, as they are able to persist and outcompete native, locally adapted species despite experiencing bottlenecks that reduce their genetic diversity, and thereby possibly their fitness^9,10^. However, there is growing evidence that genuinely paradoxical invasions are not so common^11^, as the loss of genetic diversity in invasive populations is less frequent and less intense than previously expected^12–14^. In addition, low genetic diversity in introduced populations measured at neutral markers (e.g., microsatellites) does not necessarily correlate with low variation in ecologically relevant traits^11^. Indeed, quantitative variation is usually lost at a reduced rate during invasions compared to diversity at molecular markers^15^, and pre-adaptive traits that confer success in the invaded range may render reduced genetic diversity inconsequential^16,17^. Furthermore, the degree of genetic loss may differ under distinct invasion histories. The amount of genetic diversity brought to the introduced population increases with the size of the propagule and additional re-introductions during multiple introduction events from the same or genetically distinct source populations^17^. In rare cases, genetic diversity might be higher within an introduced population than its native, source populations^18^. Sometimes, introductions originate from an already invasive population rather than a native population—a phenomenon known as the ‘*bridgehead effect’*^19–21^. This may lead to an extreme loss of diversity, as subsequent introductions arise from an already depauperate introduced population^22^. Investigating patterns of genetic diversity within the native and introduced populations of a species may provide insights into past demographic events and allow for reconstructing its invasion history^23,24^.

The Formosan subterranean termite *Coptotermes formosanus* (Shiraki) is currently recognized by the IUCN as one of the 100 worst invasive species in the world^25^, establishing invasive populations in Japan, Hawaii, and the southeastern United States^26^. Like all invasive termites, this species nests in and feeds on wood, thereby increasing its chance of being transported through merchandise trade^27^. *Coptotermes formosanus* is thought to be native to eastern Asia, though its exact origin remains unclear. It has long been suspected to originate from the vicinity of Formosa (i.e., Taiwan), where the type specimen was described^28^. A southern China origin was also suggested due to the presence of termitophilous beetles associated with *C. formosanus* colonies^29^; however, these beetles were later found to also occur within colonies in Japan^30,31^. This southern China origin was previously supported by the high diversity of *Coptotermes* species present (24 species^32^), but the recent identification of at least nine synonymized species of *C. formosanus* in the region undermines this hypothesis^33^. Recent phylogeographic studies using mitochondrial DNA (mtDNA) have also struggled to determine the origin of this species, as the variation of this marker is extremely low. These studies found either no variation between samples from Taiwan, China, and Japan^34,35^, or extremely low levels^36–38^. Even the complete mitochondrial genome reveals more than 99.9% similarity, with only a six nucleotide difference between three Japanese islands^39^. Overall, these studies have failed to conclusively identify the origin of the species within East Asia; however, they all suggest that the Chinese, Taiwanese, and Japanese populations are closely related, hinting at an early human-mediated movement of the termite throughout this region^34,35,37,38,40^.

Several studies have also attempted to reconstruct the invasion history of *C. formosanus*. However, these studies have similarly suffered from the lack of genetic variation in the mtDNA present within native populations^36,41–43^. As a result, no mtDNA variation was found in Hawaii^41^, and only 0–0.3% of variation was found on three mtDNA genes despite global sampling, with clades separated by a maximum of 3 bp differences^37^. Although the lack of mtDNA variation hampers the reconstruction of the invasion history of this species, several studies have found that introduced populations do belong to the same clade, suggesting that US populations of *C. formosanus* arise from at least two introduction events out of eastern Asia^36,42–45^. Based on microsatellite markers, at least five different sources of introduction have been suggested^46^, with high similarity between the populations of Hawaii, Louisiana, and North Carolina^44^. This finding suggests that these introduced populations either stem from a common native source population or that the mainland US population originated from a Hawaiian bridgehead. Conversely, strong differences in cuticular hydrocarbon signatures between Hawaiian and continental US samples of *C. formosanus* suggest that Hawaiian populations may not be the source of the continental US populations^47^. Overall, despite many studies attempting to elucidate this termite’s path out of eastern Asia, its exact invasion history remains unresolved.

In this study, we aimed to determine the origin(s) and the number of introduction events of *C. formosanus* out of eastern Asia and into the US. We sampled this species in both its native and introduced ranges and used double digest restriction-site associated DNA sequencing (ddRADseq^48^) to obtain markers of high resolution (i.e., single-nucleotide polymorphisms, or SNPs). We first conducted population structure and phylogenetic analyses of the global *C. formosanus* population to assess genetic structure within its native range and determine the genetic relationship between native and introduced populations. Second, we used approximate Bayesian computation (ABC) to decipher its worldwide routes of invasion. Finally, we investigated introduction-induced effects on population demography, such as population bottlenecks, expansions, migration, and admixture, to assess the consequences of the invasion on the global genetic patterns of this species.

## Results

The 359 samples yielded 0.16–43.7 million paired reads per individual, with an average of 12.9 million reads. Thirty-four individuals had a high amount of missing data (i.e., ≥30%), and were thus removed from the dataset. The final dataset contained 22,229 polymorphic loci and 33,601 SNPs for 325 individuals from the 22 populations, with an average coverage of 44× and 6.8% of missing data. To prevent linkage from affecting the population structure and phylogenetic results, only one random SNP per locus was kept. The inbreeding coefficients (*F*_IS_) as well as the observed and expected heterozygosity values for each locality are provided in Supplementary Fig. 1.

### Population structure

**Fig. 1:**
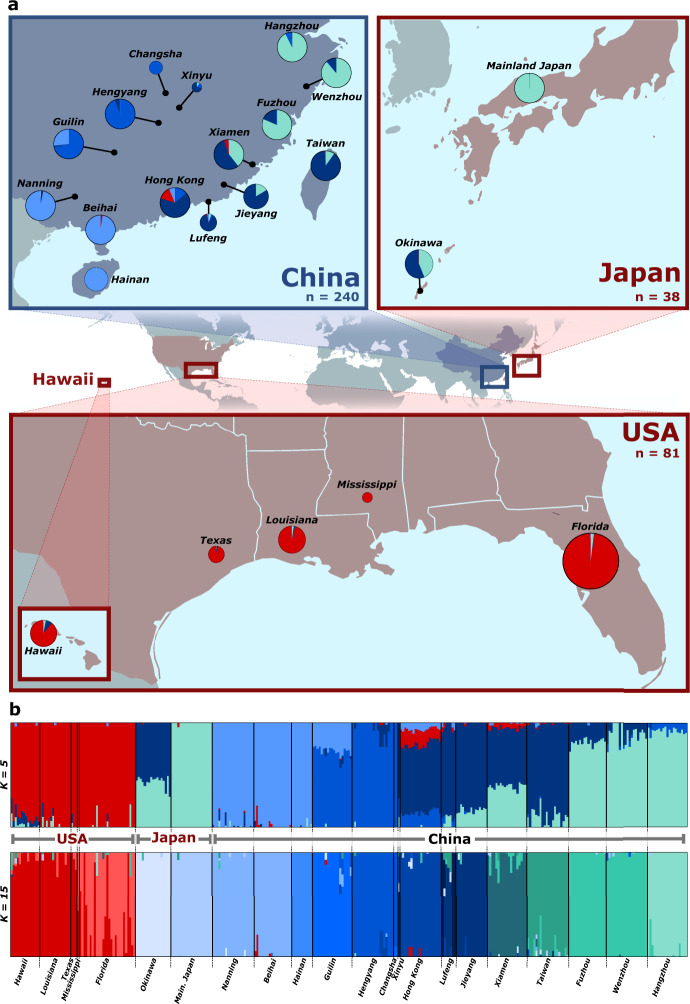
Genetic clustering of *C. formosanus* populations. **a** Pie charts of fastSTRUCTURE assignments (for *K* = 5) for each sampling location of *C. formosanus* in its native and introduced range. Pie chart size is proportional to the number of samples. **b** fastSTRUCTURE assignment for each individual sampled for *K* = 5 and 15. Each color represents a distinct genetic cluster and each vertical bar represents an individual.

Substantial structure was observed among the *C. formosanus* populations from fastSTRUCTURE, with *K* = 15 best explaining the structure in the data (Fig. 1a, b; *F*_ST_ values between all pairs of populations are supplied in Supplementary Fig. 2). At this value of *K*, 10 out of the 15 native populations represent distinct genetic clusters, with the five remaining localities mostly grouping with their geographic neighbors. Conversely, the five US states segregate into two genetic clusters, with one of the clusters comprised primarily of individuals from Florida. In addition, the populations of mainland Japan and Okinawa do not cluster together. Overall, when *K* = 15, the native and the invasive US populations share no strong ties with one another, and *K* must be decreased to five before clustering between the two becomes apparent (Fig. 1b). At *K* = 5, the entire US range clusters as one genetic entity, with its strongest tie to the native range being the Hong Kong region.

**Fig. 2:**
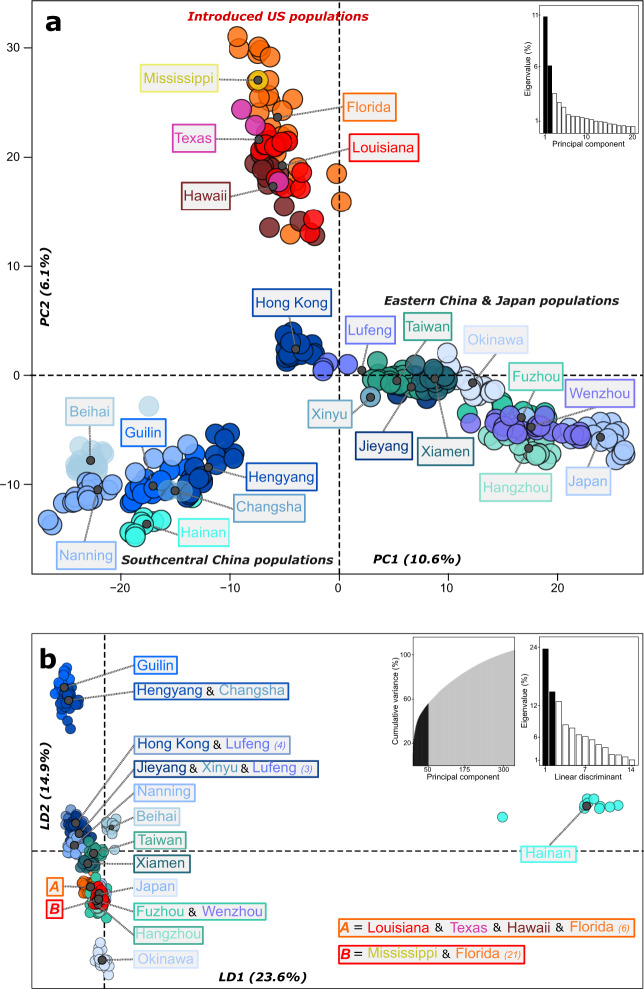
Population substructure in the global *C. formosanus* population. **a** Principal component analysis (PCA) of all *C. formosanus* individuals. The axes represent the first two principal components (PC). Only the first 20 PC’s (out of 324) are shown in the eigenvalue inset graph, with the black bars representing the two plotted PCs. **b** Discriminant analysis of principal components (DAPC) with best support for *K* = 15 genetic clusters. The axes represent the first two linear discriminants (LD). The first inset graph shows the cumulative variation explained by the PCs, with only the PCs in the black shaded area utilized for the DAPC. The second inset graph depicts the eigenvalues for all linear discriminants, with the black bars representing the two plotted LDs.

The PCA and DAPC revealed similar results to that of fastSTRUCTURE. For the PCA, samples from a given native locality mostly cluster together, suggesting that different native localities are genetically distinct from each other (Fig. 2a). Three main clusters are apparent: (1) southcentral China populations, (2) eastern China/Japan populations, and (3) introduced US populations (Fig. 2a). Again, Hong Kong and adjacent regions were most similar to the invasive US populations. The *find.clusters* function found strong support for 15 genetic clusters, with southcentral China populations again distancing themselves from eastern China/Japan and invasive US populations; however, the DAPC could not effectively distinguish between eastern China/Japan and US populations (Fig. 2b). Notably, the US invasive samples were grouped into two separate clusters: (A) one cluster including all US invasive populations (including some Florida samples, and excluding Mississippi), and (B) a second cluster including the other samples from Florida and the one sample from Mississippi. Therefore, only samples from Florida were split between the two genetic clusters.

**Fig. 3:**
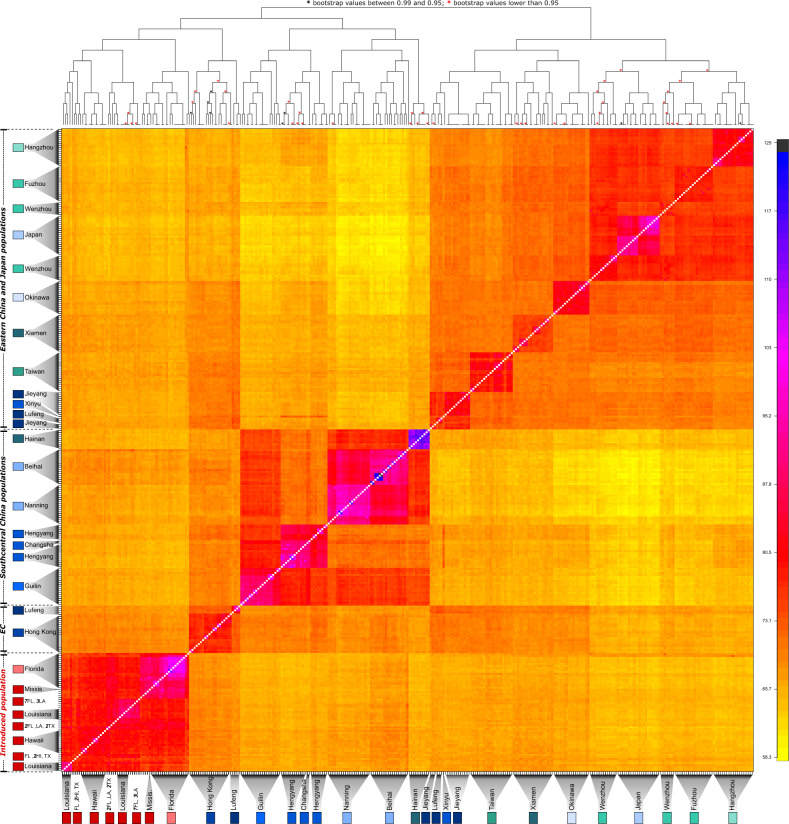
fineRADstructure analysis of *C. formosanus*. Co-ancestry matrix between each pair of individuals inferred using fineRADstructure. Each pixel represents the individual co-ancestry coefficient between two individuals. Low co-ancestry coefficient values are depicted by yellow colors, whereas high values are indicated by darker colors.

Similar patterns were identified using fineRADstructure with samples belonging to a given locality highly related to one another, indicative of the high population structure in the native range (Fig. 3). Notably, the entire US introduced population, including Hawaii, clusters together. This analysis also uncovered the three distinct clusters identified by the PCA analysis—two solely comprising geographically adjacent native regions (southcentral China populations in one cluster and eastern China/Japan populations in the other) and one grouping the entire US invasive region with Hong Kong (Fig. 3).

### Phylogenetic analysis

**Fig. 4:**
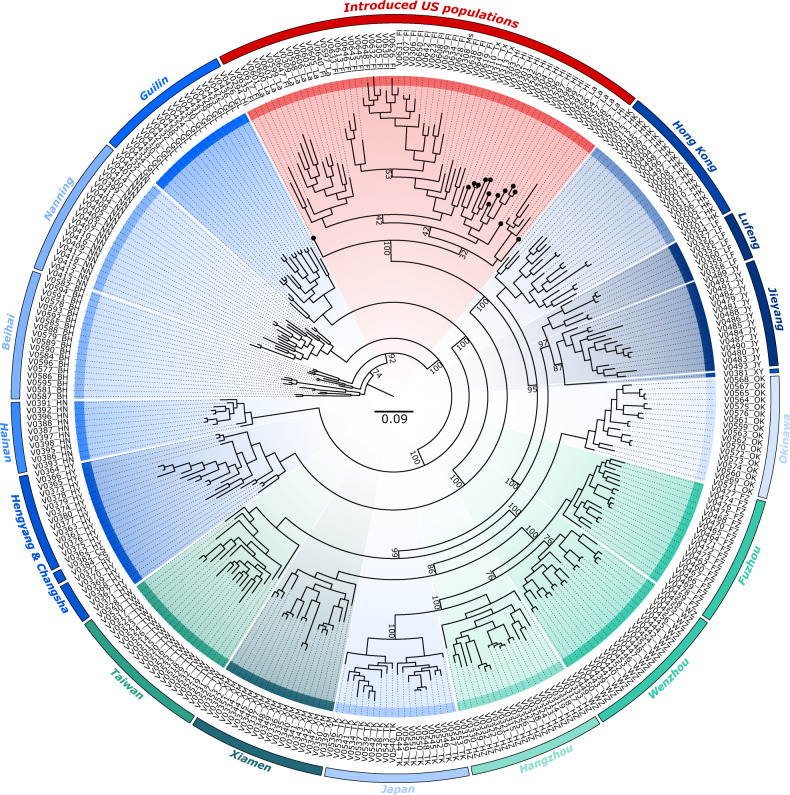
Maximum likelihood phylogenetic tree of *C. formosanus*. The sampling locations are colored according to their fastSTRUCTURE assignments (*K* = 15). For clarity, bootstrap values are only indicated for major branching events. Samples from Hawaii are highlighted with a round tip.

We constructed ML phylogenies for the full set of individuals using a further refined dataset of SNPs to determine if there were any strongly supported phylogenetic lineages. The 22,229 unlinked SNPs were stripped of invariant sites, leaving 21,542 SNPs to construct the tree. The MRE-based bootstopping criterion was satisfied by 400 bootstrap replicates, with the best-scoring likelihood and majority rule extended consensus trees for the SNP dataset having a middling amount of support throughout the topology; however, the tree was consistent with results from the clustering analyses. First, the strong population structure in the native range is again apparent as almost every native population represents its own branch of the tree (Fig. 4). In addition, the invasive US populations fall out as a single clade and appear most closely related to Hong Kong (Fig. 4). Interestingly, samples from Hawaii cluster at the base of this “introduced” branch, despite the presence of five Louisianan samples segregated within the Hawaiian samples.

### Invasion history

The first step of the ABC analysis found the most support for the introduced US population originating from admixture between southcentral and eastern Asia scenario (S1c; 375 out of 500 RF votes), rather than from southcentral China (46 RF votes) or eastern Asia (79 RF votes) exclusively. For the second step, the Hong Kong region (i.e., Hong Kong, Jieyang, Lufeng, Okinawa, Taiwan, and Xiamen), was separated from eastern Asia (i.e., sub-eastern Asia—Fuzhou, Hangzhou, mainland, and Wenzhou), with the introduced US population best explained by admixture between eastern China and Hong Kong (S2c) when considering only two-population admixture. However, this scenario was outvoted (only 31 RF votes) in the third step when the possibility of admixture between all three populations was considered, regardless of the first admixture event (S3b, c and d; 469 cumulative RF votes). In addition, sub-steps 2A and 2B confirmed the inclusion of the Japanese populations into the two eastern Asian sub-regions.

When the Hong Kong region was reintegrated within eastern Asia, the fourth step (analyzing Hawaii separately from the mainland US) was not conclusive, as two scenarios gained a similar number of RF votes. The first one suggested that Hawaii and the US mainland originated independently from eastern Asia and southcentral China, respectively (S4c; 155 RF votes), while the other proposed that Hawaii results from admixture between eastern Asia and southcentral China, with the US mainland arising from a Hawaiian bridgehead (S4g; 136 RF votes). This discrepancy seemed to be driven by a split between most Florida samples and the rest of the mainland, which is also depicted in fastSTRUCTURE. Indeed, sub-step 4 subsequently confirmed a Hawaiian bridgehead to the US mainland (to Louisiana/Texas; Sub4c; 194 RF votes), when the US mainland was split between Florida and Louisiana/Texas. The fifth step confirmed that both eastern Asian regions were involved in the invasion of Hawaii and Louisiana/Texas (S5c; 291 RF votes), when the Hong Kong region was separated from eastern Asia (i.e., sub-eastern Asia).

**Fig. 5:**
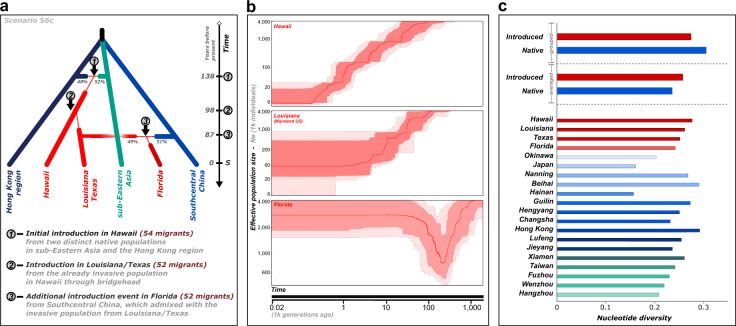
The invasion history of *C. formosanus*. **a** Graphical representation of the most likely invasion history scenario for *C. formosanus* out of Asia tested through ABC RF. Thin dotted lines represent bottleneck events. Estimated duration of each bottleneck event, as well as all of the posterior parameter estimates are provided in Supplementary Table 11. Time is not to scale, with ***S*** indicating sampling time. **b** Estimation of the variation of effective population size through time for three invasive localities using Stairway Plot 2. The solid red line is the estimate of the median effective population size, and the light and dark red shaded areas represent the 95% and 75% confidence intervals, respectively. **c** Nucleotide diversity in the introduced and native range. All native and introduced localities were first analyzed while grouped together, then independently.

Finally, the sixth and final step revealed that the population in Florida most likely resulted from admixture between Louisiana/Texas (49%) and southcentral China (51%) 87 years ago, and Louisiana/Texas to originate solely from the admixed Hawaiian bridgehead population 98 years ago (Fig. 5a; S6c; 220 RF votes). Also, the first introduction to Hawaii was estimated to have occurred 138 years ago from admixture between the Hong Kong region (48%) and sub-eastern Asia (52%). A detailed description of the step-by-step ABC RF analysis, including the priors used for each step, comparative scenario statistics and the posterior parameter estimates for the final invasion model (S6c) is available in Supplementary Tables 1–11, Supplementary Figs. 3–10, and the Supplementary Methods.

### Demographic history

The demographic history of each population was inferred through Stairway Plot 2, using an average of 14,138 SNPs per population after SNPs with missing data were filtered out. Distinct demographic histories were present in both the native and the introduced US ranges (Fig. 5b). Most native populations have experienced a gradual decline in their effective population size, while Fuzhou, Hangzhou, Jieyang, and the two Japanese populations experienced a bottleneck followed by a period of rapid growth. In the US populations, Hawaii and Louisiana both underwent a gradual decline in their effective population size, while Florida experienced a bottleneck followed by a period of rapid growth (Fig. 5b), corroborating the ABC results of an additional and distinct introduction event within Florida.

## Discussion

Our study unravels the global invasion history of *C. formosanus*, retracing its invasion pathway out of eastern Asia and assessing introduction-induced effects on its population demography and genetic diversity. Our findings reveal that the global distribution of *C. formosanus* has been shaped by multiple introductions out of eastern Asia, coupled with a bridgehead event. The complex invasion history of *C. formosanus* began with an initial introduction in Hawaii (~1870) that originated from at least two distinct events, and their admixture, out of sub-eastern Asia and the Hong Kong region. This introduced Hawaiian population later served as the source for the invasion of the US mainland (~1930), where an additional introduction event from southcentral China occurred in Florida (~1940). These dates match up well with the generally accepted timeline of its invasion to the US. The first recorded observation of this species in the US dates back to the early 1900s in Hawaii^49,50^, although there is evidence that it had been established as early as 1869^51^. Within the mainland US, *C. formosanus* was first recorded in South Carolina (1957^52^), Texas (1965^53^), and Louisiana (1966^54^). However, its strong association with military ports receiving and storing equipment and supplies from the Pacific theater after World War II^54^ led to the widely held theory that it was introduced to the US mainland around this time period, aligning closely with our time estimates.

Much of the diversity present in the native range is highly structured among the native populations, with almost every native population representing a unique genetic cluster. The PCA and low values of K revealed two primary groups within the sampled Chinese range (eastern and southcentral China). Such a split is found in other eastern Chinese organisms^55,56^, and has been proposed to be a relic of the Last Glacial Maximum that would have restricted available habitat for subtropical species to the extreme southern edge of China^57^.

There was a slight reduction of genetic diversity within the introduced US range compared to the entire native range. Yet, the genetic diversity within each US population was equivalent to the diversity present in each of the native populations, indicating this termite’s invasion has not been accompanied by a drastic loss of diversity at the population level (Fig. 5c). This finding differs from a previous study comparing the diversity of *C. formosanus* between its native and introduced range using microsatellite markers, which found substantially reduced diversity in each introduced population sampled relative to the native range^46^. However, RADseq derived SNPs have been found to more accurately estimate genome-wide diversity than microsatellites^58,59^, which may explain the contrasting results obtained in the present study. This outcome is perhaps surprising because the founding event following an introduction usually reduces diversity within invasive populations, suggesting that multiple introduction events from distinct source populations may have prevented and even reversed the loss of diversity within the invasive range of *C. formosanus*.

Our clustering, phylogeographic and ABC results consistently show that eastern China is the prominent source of the invasive populations in Hawaii and the mainland US, congruent with the hypothesis of Husseneder et al.^46^. Interestingly, Husseneder et al.^46^ also identified two genetic clusters within the US mainland, with South Carolina being distinct from Louisiana and North Carolina. Unfortunately, samples from South Carolina were not included in our analyses, preventing the identification of a possible link between South Carolina and the second cluster we found in Florida. In addition, we are unable to rule out a Japanese influence in the *C. formosanus* invasion of the US, and whether the Japanese populations^60^ are invasive. While the demographic histories for both Okinawa and mainland Japan did display evidence of a bottleneck, genetic diversity statistics of the two populations were not drastically different from Chinese populations and clustering between the two is not present unless *K* is substantially reduced.

Human and merchandise transportation hubs have been shown to be an important factor in the spread of invasive species throughout the world^3,5,61,62^, and eastern Asia includes some of the largest and busiest ports in the world, such as Hong Kong, Shanghai, and Tokyo. The long history of both trade and immigration between China and the Kingdom of Hawaii dates back to the late 1700s, and centers around the southeastern region of China (i.e., Hong Kong and neighboring areas) and Honolulu (main port of Hawaii)^63,64^. Notably, the population of Chinese in Hawaii drastically increased from 364 individuals in 1852 to 18,254 individuals in 1884^63^, with most immigrants originating from this southeastern portion of China. Japan has also had a long history of immigration to Hawaii, from both the mainland^65^ and Okinawa^66^, as almost 200,000 Japanese moved to Hawaii between 1886 and 1924^65^. These large-scale immigration events from eastern Asia coincide with the first suspected evidence of a subterranean termite in Hawaii^51^.

Our ABC analysis suggests that the US mainland populations of *C. formosanus* likely arose from an already established invasive population in Hawaii through bridgehead rather than from an independent introduction directly from the native range. Indeed, cases of introduced populations themselves becoming the source of further introductions are being recognized more commonly^67–71^, including in other eusocial insects like invasive ants. For example, global phylogeographic analysis of the red imported fire ant *Solenopsis invicta* revealed that after its primary introduction event into the southeastern US from South America, this southeastern US population served as the source for its further spread to the rest of the world^20,72^. Furthermore, ants as a whole display striking secondary introduction rates, with over 75% of ants intercepted at US and New Zealand ports of entry originating from locations where they had already been introduced^21^. While termite interceptions at US ports of entry have hinted at their potential to spread via bridgeheads^73^, our study empirically elucidates a bridgehead invasion in a non-ant social organism, with *C. formosanus* utilizing Hawaii as a stepping-stone for its subsequent invasion of the US mainland. This suggests that bridgeheads may play a crucial role for social insects in achieving multi-continental distributions, warranting further research into the invasion histories of other globally distributed social insects. For example, the West Indian drywood termite *Cryptotermes brevis* (Walker) likely represents a bridgehead invader, as it is native to the coastal deserts of Peru and Chile and is now invasive on five continents^27^.

Bridgehead introductions have drastic effects on genetic diversity as introduced populations often experience bottleneck events. While some invasive species merely tolerate this genetic depletion, some benefit from the periodic purge of deleterious alleles through founder effects^12,74–76^. In this context, bridgehead populations may reduce inbreeding depression in subsequent invasive populations through the purge of deleterious alleles during recurrent founder effects^77^. On the other hand, reduced genetic diversity in bottlenecked bridgehead populations may promote the rapid evolution of invasive traits, as rates of adaptive evolution substantially increase with reductions in population size^78,79^. For this reason, bridgeheads have been hypothesized to be a stepping-stone for invasion by selecting for invasive traits^19,20,80,81^. These traits may increase the ability of an invader to be further spread to novel locations, confer greater ecological advantage that enables them to outcompete native species, and aid in circumventing the low genetic diversity in bottlenecked populations^82–86^. Despite this hypothesis of adaptive spread as a driver of the bridgehead effect, empirical evidence for this evolution of invasiveness is still lacking^22^, and the evolution of specific invasive traits within the Hawaiian population of *C. formosanus* remains undetected.

The presence of two genetic clusters in the mainland US signaled that the invasion pattern was more complex than just a single introduction from the Hawaiian bridgehead, and subsequent analysis confirmed a separate introduction event from southcentral China had indeed occurred. It seems most likely this additional event occurred within Florida, given it clustered separately from the other US populations and that the scenario describing Florida as a result of admixture between Louisiana/Texas and southcentral China was found most probable, as well as its unique demographic history. We also considered the possibility of interspecies admixture being the cause of Florida clustering separately, as a sister species of *C. formosanus*, *C. gestroi* (Wasmann), is also established in Florida^87^. These species have overlapping nuptial flights^88^ and form tandem pairs of reproductive individuals^89,90^; however, this hypothesis was ultimately found to be unlikely, as hybridization should be identifiable at low values of *K*. Furthermore, these two species are also sympatric in Hawaii and Taiwan, and none of the three regions display a highly negative *F*_IS_ commonly observed due to hybridization. Instead, Florida as a separate genetic cluster appears to stem from a distinct introduction event out of southcentral China.

Multiple events out of the native range from different source populations differ from the invasion pattern observed in another invasive subterranean termite, *Reticulitermes flavipes*. This species is native to the eastern US and has been introduced to France, Canada, the Bahamas, Uruguay, and Chile. Interestingly, most introduced populations of this species seem to originate specifically from New Orleans, Louisiana^91,92^. While New Orleans is an important hub for global trade, this species is also present in major trading cities along the eastern seaboard that have seemingly played no role in their spread. This suggests that certain traits of *R. flavipes* colonies within the New Orleans region may have pre-adapted this population to invasion, such as their distinct breeding structure and reduced antagonism between non-nestmates^92,93^, which they share with introduced populations in France^92,94–97^ and Chile^92^. Therefore, this finding is similar to the hypothesis suggested for bridgehead populations, whereby the evolution of specific traits conferring higher invasiveness primes a population for further invasion. This similar scenario has been coined the ‘*Anthropogenically Induced Adaptation to Invade’* and suggests the evolution of adaptations to human-modified habitats in specific native populations favor their subsequent spread^16^. Such local pre-adaptation to invasion has been observed in the native range of the little fire ant *Wasmania auropunctata*, with natural populations mostly displaying small non-dominant colonies headed by sexually produced reproductives, while anthropogenic populations shift to large and dominant supercolonies headed by clonal reproductives^98^. The similar life‐histories of native anthropogenic populations and invasive populations suggest that these traits, which evolved within its native range, may act as pre-adaptations to human-altered habitats and favor its worldwide invasion^16^. Yet, despite being one of the most widespread invasive termites worldwide, introduced colonies of *C. formosanus* do not appear to have experienced a major shift in their breeding system or colony structure when compared to native colonies^46,99,100^. Therefore, the worldwide invasion of this termite seems unrelated to these life-history traits. However, as native samples in this study were collected solely from human-disturbed habitats, we cannot be certain *C. formosanus* has not already undergone selection toward anthropogenic landscapes in their native range, like *W. auropunctata*. Whether there are other physiological factors enhancing their ability to thrive in human-modified habitats or whether no specific pre-adaptation is required, meaning each native population has the capacity to produce an invasion viable propagule, remains to be seen. Overall, these findings stress the need for comparative research between the introduced and native range, where key evolutionary processes promoting invasions may be occurring.

## Methods

### Sample collection and molecular methods

*Coptotermes formosanus* colonies were sampled in both their native and introduced ranges (Supplementary Table 12), with workers stored in 100% ethanol for subsequent sequencing. In the native range, colonies were sampled across thirteen localities in mainland China (southcentral China—Beihai, Changsha, Guilin, Hainan, Hengyang, Nanning, and Xinyu; eastern China—Fuzhou, Hangzhou, Jieyang, Lufeng, Wenzhou, and Xiamen), Hong Kong, and Taiwan. In the introduced range, colonies were sampled in mainland Japan and Okinawa, as well as in Hawaii, Texas, Louisiana, Mississippi, and Florida. Total genomic DNA of 359 workers was extracted following a modified Gentra Puregene extraction method (Gentra Systems, Inc., Minneapolis, MN, USA), then libraries were prepared and sequenced at the Texas A&M AgriLife Genomics and Bioinformatics Service facility following the protocol of Peterson et al.^48^. Briefly, genomic DNA was first digested with the restriction enzymes *SphI* and *EcoRI*. Following restriction digestion, each sample was ligated with unique indexed adapters. Then, samples were PCR amplified with iProof™ High-Fidelity DNA Polymerase (Bio-Rad), and purified using AMPure XP beads (Beckman Coulter Inc.) to make the ddRADseq library. Each library pool was size selected to a range of 300–500 bp using the BluePippin system (Sage Science Inc.). Quantity and size distribution were assessed using the Qubit® 2.0 Fluorimeter (Life Technologies Corp.) and Bioanalyzer 2100 System (Agilent Technologies). Amplified fragment libraries were then pooled in equimolar amounts and sequenced on six lanes of an Illumina HiSeq 2500 machine to generate 150 bp pair‐end reads.

### Raw read quality filtering and processing

Raw sequences for each lane were examined separately to check for read quality and adapter contamination using FastQC v0.11.8^101^, with reads of the two lanes then concatenated after ensuring no lane discrepancies (i.e., R1’s & R2’s combined separately). Forward and reverse reads were assembled and SNPs were generated using the de novo pipeline of Stacks v.2.41^102^. The main parameters for the analysis were optimized following the *r80 loci* method^103^. Briefly, a representative subset of samples was taken from the main dataset to run through the de novo pipeline under varying values of its most influential parameter (-M, the number of mismatches allowed between putative alleles), in order to identify the value that produced the greatest number of polymorphic loci found in 80% of the population. After parameter optimization, filtered reads were run through the de novo pipeline of Stacks, which built and genotyped the paired-end data, as well as called SNPs using the population-wide data per locus. Only SNPs present in at least half of the individuals in all populations were kept for downstream analyses. In addition, alleles at low frequency (<0.05) and loci with high heterozygosity (>0.7) were filtered out as these are likely byproducts of sequencing errors and paralogs^104^. Furthermore, SNPs with <5× mean coverage and exceeding 200× mean coverage were filtered out using VCFtools v.0.1.15^105^, to buffer against unlikely SNPs and avoid highly repetitive regions of the genome. To prevent linkage disequilibrium (LD) between SNPs from affecting the population structure and phylogenetic analyses, only one random SNP per locus was kept. All subsequent file format conversions were accomplished through PGDSpider v.2.1.1.5^106^.

### Genetic diversity and population structure

Genetic diversity (expected heterozygosity (H_E_), observed heterozygosity (H_O_), inbreeding coefficients (*F*_IS_)), and population differentiation (*F*_ST_) indices for each locality were calculated in Stacks. Population structure among the 22 sampled locations was analyzed using three complementary approaches. First, population structure was assessed by estimating the most likely number of genetic clusters (i.e., *K*) in the dataset using fastSTRUCTURE v1.040^107^. fastSTRUCTURE runs were parallelized and automated using Structure_threader^108^. Different values of K ranging from 1 to 22 were analyzed, and the best value was selected using the *chooseK.py* function from the fastSTRUCTURE package. Plots were created by Distruct v2.3^109^ (available at http://distruct2.popgen.org). Second, we used both a principal component analysis (PCA) and discriminant analysis of principal components (DAPC) to estimate clustering in the data (see Supplementary Methods; Supplementary Figs. 11–12). DAPC describes clusters in genetic data by creating synthetic variables (discriminant functions) that maximize variance among groups while minimizing variance within groups^110^. We first performed the PCA, then ran the *find.clusters* clustering algorithm using the PCA results to infer the most likely number of genetic groups, as DAPC requires prior groups to be defined. The Bayesian information criterion was used to select the most likely number of genetic clusters. Finally, the function *optim.a.score* to identify the optimal number of principal components to inform the DAPC, as too few components could hinder discriminatory power between groups, while too many could lead to overfitting. Both the PCA and DAPC were run in R^111^ through the *adegenet* package^112^. Third, we used the program fineRADstructure v0.3.2^113^ to infer population structure via shared ancestry among *C. formosanus* individuals. Modified from fineSTRUCTURE^114^, fineRADstructure is specifically designed for RADseq data, and does not require information about location of loci on chromosomes or phased haplotypes. Loci were first reordered according to LD, as strong LD combined with unsorted loci could result in an overconfident clustering of individuals. A co-ancestry matrix was then constructed from the sorted loci and individuals were assigned to populations with a burn-in period of 100,000 and 100,000 Markov chain Monte Carlo iterations. Finally, a tree was constructed from the default parameters, and results were visualized in R through scripts provided with the program (available at http://cichlid.gurdon.cam.ac.uk/fineRAD structure.html).

### Phylogenetic analysis

Maximum likelihood (ML) phylogeny among *C. formosanus* individuals was inferred using RAxML v8.2.12^115^. We applied an acquisition bias correction to the likelihood calculations as the alignments were composed exclusively of SNPs^116^, removing all invariant sites in the alignments with the Phrynomics R script (available at https://github.com/bbanbury/phrynomics/). We then conducted a rapid bootstrap analysis and search for the best-scoring maximum likelihood tree using the extended majority rule (MRE)-based bootstopping criterion^117^ to determine an appropriate amount of bootstrap replicates. All searches were performed using the GTR + G nucleotide substitution model.

### Invasion history

We inferred the invasion routes and colonization history of *C. formosanus* by selecting the most likely evolutionary scenario using ABC^118^. The number of competing scenarios exponentially increases with the number of potential source populations and demographic events compared in the analysis^19,24^, which requires considerable computational effort. Therefore, to more efficiently allocate this effort, we utilized a recently developed random forests (RF) machine learning tool to conduct model selection and parameter estimation (ABC RF^119^). ABC RF requires a considerably reduced number of simulated datasets compared with alternative methods, while also providing a more reliable estimate of the posterior probability for the best model. We also decreased the required computational effort by inferring the invasion history of *C. formosanus* through a step-by-step analysis (six different steps), which is commonly performed in ABC studies^67,120,121^. The mainland Japan and Okinawa populations were included in the ABC analysis as a member of the eastern Asia region given their strong clustering within the region in the population genetic and phylogenetic analyses (see “Results”). In addition, two localities (Xinyu and Mississippi) were excluded from all ABC steps as only one sample was available for each location. Briefly, the first step aimed at identifying which region(s) of the native range (i.e., eastern Asia, southcentral China, or an admixture of both) have contributed to the introduction of *C. formosanus*, with the introduced US range pooled as a single population. The second and third steps aimed at determining which region(s) of eastern Asia (i.e., only the Hong Kong region, only the other localities within eastern Asia, or an admixture of both) played a role. The fourth and fifth steps tested for the origin of the Hawaiian population and the possibility of a bridgehead effect in Hawaii; thus, Hawaii was analyzed separately from the mainland US. Finally, the sixth step considered the occurrence of a distinct introduction event to Florida.

Model simulations were first run in DIYABC v2.0^122^, with at least 10,000 simulations per model performed on 2000 randomly sampled SNPs for each of the steps above. Priors were set uniform for all model parameters and selected based on historical records. The timing of introduction events to the US was set to between 50 and 300 years ago, with the condition that the introduction in Hawaii (for steps 4–6) occurred prior to the introduction to the southeastern US, consistent with historical records^46^. In addition, for all scenarios tested, the decrease in effective size of an introduced population was allowed to vary between 1 and 100 migrants, and the duration of the bottleneck set to vary between 0 and 50 years for each introduction event. The range of all other priors was adjusted by evaluating the posterior distributions of the preliminary simulated datasets, then setting the prior distribution as wide as possible while retaining biological meaning. All summary statistics included in the DIYABC software were used for each analysis, and both model selection and parameter estimation were performed through ABC RF^119,123^, available in the *abcrf* R package.

### Exploring changes in population sizes

We inferred the demographic history of each locality by using Stairway Plot 2^124^ to investigate recent changes in population size (e.g., bottleneck, expansion, admixture, etc.). Unlike traditional skyline plot methods for demographic inference which compute a likelihood for a whole sequence^125^, Stairway Plot 2 instead calculates the composite likelihood of a given SNP frequency spectrum (SFS)^126,127^. This method uses the expected number of mutation(s) per base pair to measure time and *θ* per base pair to measure population size (*θ* = 4*N*_e_*µ*, where *N*_e_ is the effective population size and *µ* is the mutation rate per generation). The full catalog of SNPs was retained for this analysis; however, only SNPs with no missing data (by population) were used in the SFS calculations due to the difficulty of integrating missing data when modeling the SFS under coalescent approaches. Folded SFSs for each locality were generated by the *vcf2sfs* R script^128^. The demographic history for each population can be seen in Supplementary Fig. 13.

### Statistics and reproducibility

Sampling locations and sample sizes for each location are listed in Supplementary Table 12. More detailed descriptions of the ABC and DAPC processes are available in the Supplementary Methods.

### Reporting summary

Further information on research design is available in the Nature Research Reporting Summary linked to this article.

## Supplementary information

Supplementary Information

Supplementary Data

Reporting Summary

## Data Availability

Raw sequence files are deposited at the National Center for Biotechnology Information under BioProject accession number PRJNA666619. In addition, SNP data (.vcf) can be downloaded from the Open Science Framework database, https://osf.io (10.17605/OSF.IO/QSBD5).
