# Supplementary material for: Bridgehead effect and multiple introductions shape the global invasion history of a termite
Source: Commun Biol. 2021 Feb 12;4:196. doi: 10.1038/s42003-021-01725-x (PMC7881189; doi:10.1038/s42003-021-01725-x)
Supplement: Supplementary file 1 — Supplementary Information [file 42003_2021_1725_MOESM1_ESM.pdf]

Supplementary information for 'Bridgehead effect and multiple introductions shape the global invasion history of a termite'

Alexander J. Blumenfeld[1,†,*], Pierre-André Eyer[1,†], Claudia Husseneder[2], Jianchu Mo[3], Laura N.L. Johnson[1,4], Changlu Wang[5], J. Kenneth Grace[6], Thomas Chouvenc[7], Shichen Wang[8], Edward L. Vargo[1]

[1]Department of Entomology, 2143 TAMU, Texas A&M University, College Station, Texas, 77843-2143, USA

[2]Department of Entomology, Louisiana State University Agricultural Center, Baton Rouge, LA 70803, USA

[3]Ministry of Agriculture Key Lab of Molecular Biology of Crop Pathogens and Insect Pests, Institute of Insect Sciences, College of Agricultural and Biotechnology, Zhejiang University, Zhejiang, PR China

[4]Department of Veterinary Sciences, University of Wyoming, Laramie, Wyoming, 82070, USA

[5]Department of Entomology, Rutgers, The State University of New Jersey, New Brunswick, NJ 08901, USA

[6]Department of Plant and Environmental Protection Sciences, University of Hawaii at Manoa, Honolulu, HI, USA

[7]Entomology and Nematology Department, Fort Lauderdale Research and Education Center, Institute of Food and Agricultural Sciences, University of Florida, Fort Lauderdale, FL, USA

[8]Texas A&M AgriLife Genomics and Bioinformatics Service, College Station, TX, USA

[†] Alexander J. Blumenfeld and Pierre-André Eyer should be considered joint first authors

*Corresponding author:
Alexander J. Blumenfeld
Department of Entomology,
Texas A&M University,
College Station, 77843, Texas, USA
e-mail: alex93@tamu.edu

Supplementary Information

Supplementary Figures 1 – 13

Supplementary Tables 1 – 12

Supplementary Methods: A detailed description of the step-by-step ABC RF analysis is covered, including: 1) priors used for the analyses, 2) graphical representation and random forest votes for each scenario within each step, 3) an overall linear discriminant analysis (LDA) of the simulated datasets for every scenario for each step and 4) parameter estimates for the final invasion model. Additionally, output from the functions *find.clusters* and *optim.a.score* from our DAPC analysis is provided.

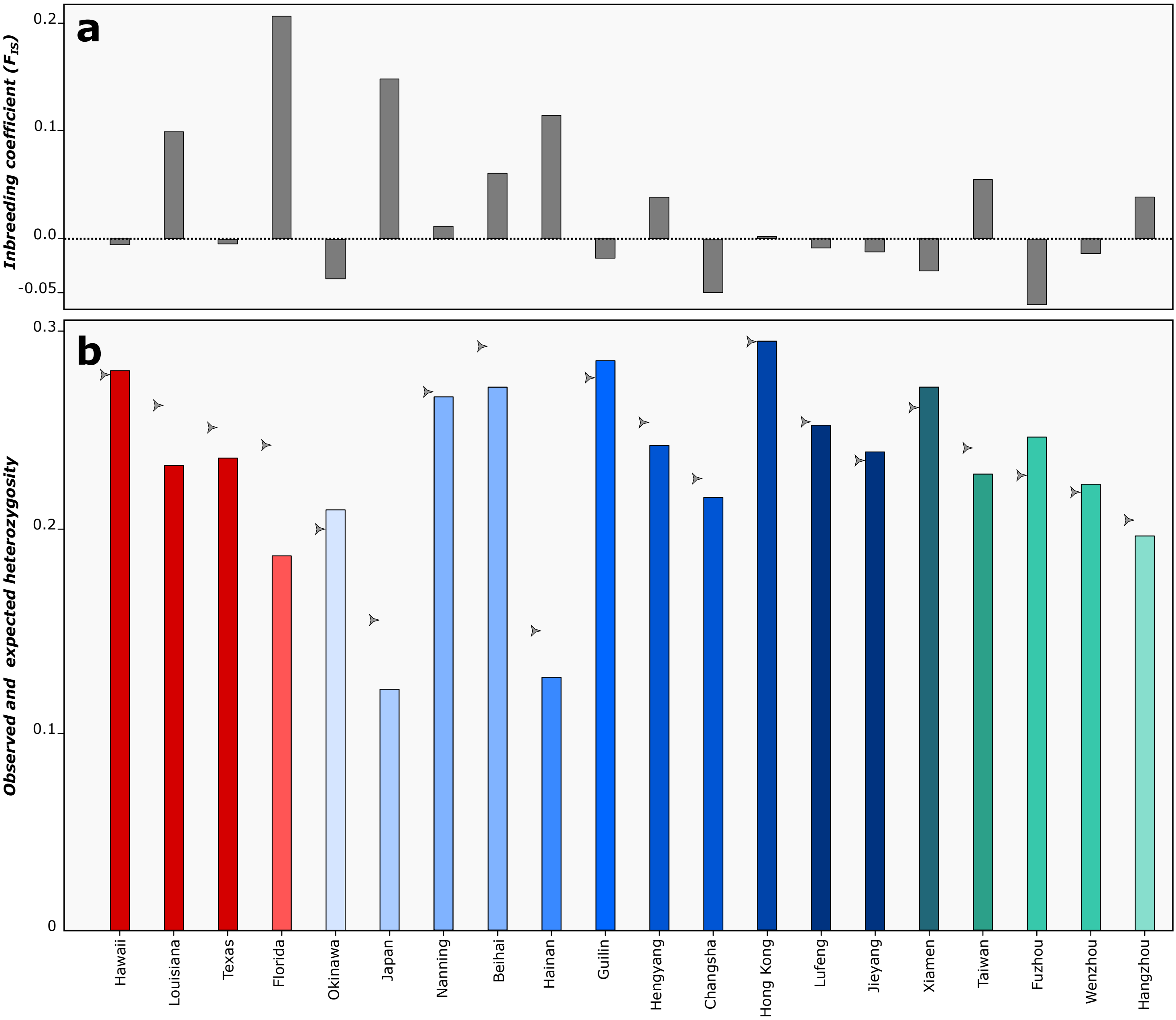

Supplementary Figure 1: **Genetic diversity statistics. a** Inbreeding coefficients (F_{IS}) for each population. **b** Mean level of observed heterozygosity for each population. Arrows indicate the expected level of heterozygosity for each population. Each population is colored according to its genetic assignment using fastSTRUCTURE (for K = 15).

	Hawaii	Louisiana	Texas	Mississipi	Florida	Okinawa	Japan	Nanning	Beihai	Hainan	Guilin	Hengyang	Changsha	Xinyu	Hong Kong	Lufeng	Jieyang	Xiamen	Taiwan	Fuzhou	Wenzhou	Hangzhou
Hawaii		0.18	0.16	0.27	0.19	0.19	0.21	0.14	0.14	0.17	0.14	0.16	0.21	0.26	0.19	0.17	0.17	0.16	0.22	0.17	0.18	0.16
Louisiana	0.18		0.09	0.13	0.04	0.11	0.06	0.12	0.11	0.03	0.11	0.11	0.19	0.20	0.12	0.10	0.10	0.08	0.14	0.10	0.10	0.07
Texas	0.16	0.09		0.19	0.09	0.07	0.13	0.11	0.11	0.08	0.10	0.09	0.17	0.16	0.08	0.06	0.05	0.05	0.10	0.06	0.06	0.05
Mississipi	0.27	0.13	0.19		0.10	0.20	0.14	0.22	0.22	0.13	0.21	0.21	0.28	0.28	0.21	0.20	0.19	0.18	0.23	0.20	0.20	0.18
Florida	0.19	0.04	0.09	0.10		0.11	0.07	0.12	0.12	0.04	0.12	0.12	0.19	0.20	0.12	0.10	0.10	0.08	0.14	0.10	0.11	0.08
Okinawa	0.19	0.11	0.07	0.20	0.11		0.14	0.14	0.15	0.10	0.13	0.11	0.19	0.17	0.07	0.08	0.07	0.06	0.09	0.06	0.06	0.08
Japan	0.21	0.06	0.13	0.14	0.07	0.14		0.15	0.15	0.06	0.15	0.15	0.22	0.23	0.16	0.13	0.14	0.12	0.18	0.14	0.15	0.11
Nanning	0.14	0.12	0.11	0.22	0.12	0.14	0.15		0.05	0.11	0.07	0.10	0.16	0.23	0.14	0.12	0.12	0.11	0.18	0.13	0.13	0.09
Beihai	0.14	0.11	0.11	0.22	0.12	0.15	0.15	0.05		0.10	0.07	0.10	0.16	0.24	0.14	0.12	0.12	0.11	0.18	0.13	0.14	0.08
Hainan	0.17	0.03	0.08	0.13	0.04	0.10	0.06	0.11	0.10		0.10	0.10	0.18	0.20	0.11	0.09	0.09	0.07	0.13	0.09	0.10	0.06
Guilin	0.14	0.11	0.10	0.21	0.12	0.13	0.15	0.07	0.07	0.10		0.06	0.12	0.20	0.12	0.10	0.10	0.09	0.16	0.11	0.12	0.07
Hengyang	0.16	0.11	0.09	0.21	0.12	0.11	0.15	0.10	0.10	0.10	0.06		0.08	0.17	0.11	0.10	0.10	0.09	0.16	0.11	0.11	0.07
Changsha	0.21	0.19	0.17	0.28	0.19	0.19	0.22	0.16	0.16	0.18	0.12	0.08		0.24	0.18	0.17	0.18	0.17	0.22	0.19	0.19	0.16
Xinyu	0.26	0.20	0.16	0.28	0.20	0.17	0.23	0.23	0.24	0.20	0.20	0.17	0.24		0.18	0.16	0.14	0.17	0.20	0.17	0.17	0.17
Hong Kong	0.19	0.12	0.08	0.21	0.12	0.07	0.16	0.14	0.14	0.11	0.12	0.11	0.18	0.18		0.10	0.08	0.06	0.06	0.04	0.04	0.10
Lufeng	0.17	0.10	0.06	0.20	0.10	0.08	0.13	0.12	0.12	0.09	0.10	0.10	0.17	0.16	0.10		0.05	0.06	0.12	0.08	0.08	0.06
Jieyang	0.17	0.10	0.05	0.19	0.10	0.07	0.14	0.12	0.12	0.09	0.10	0.10	0.18	0.14	0.08	0.05		0.05	0.10	0.06	0.06	0.06
Xiamen	0.16	0.08	0.05	0.18	0.08	0.06	0.12	0.11	0.11	0.07	0.09	0.09	0.17	0.17	0.06	0.06	0.05		0.08	0.04	0.04	0.06
Taiwan	0.22	0.14	0.10	0.23	0.14	0.09	0.18	0.18	0.18	0.13	0.16	0.16	0.22	0.20	0.06	0.12	0.10	0.08		0.05	0.05	0.12
Fuzhou	0.17	0.10	0.06	0.20	0.10	0.06	0.14	0.13	0.13	0.09	0.11	0.11	0.19	0.17	0.04	0.08	0.06	0.04	0.05		0.02	0.08
Wenzhou	0.18	0.10	0.06	0.20	0.11	0.06	0.15	0.13	0.14	0.10	0.12	0.11	0.19	0.17	0.04	0.08	0.06	0.04	0.05	0.02		0.08
Hangzhou	0.16	0.07	0.05	0.18	0.08	0.08	0.11	0.09	0.08	0.06	0.07	0.07	0.16	0.17	0.10	0.06	0.06	0.06	0.12	0.08	0.08	

***Supplementary Figure 2*: Genetic differentiation.** Matrix of F_{ST} values for each pair of populations. Each population is colored according to its genetic assignment using STRUCTURE (for K = 15).

ABC RF analysis

Supplementary Table 1: Priors used for each step of the ABC analysis.

Step(s)	Parameter	Lower bound	Upper bound
All	N_i[1]	100	10000
	N_ib[2]	1	100
	db_i[3]	0	50
	ra_i[4]	0.001	0.999
1,2	t1[5]	50	300
	t2	300	10000
3	t1	50	300
	t2	50	300
	t3	300	10000
4,5	t1	50	150
	t2	70	300
	t3	300	10000
6	t1	50	150
	t2	50	150
	t3	70	300
	t4	300	10000

[1]Effective population size.

[2]Effective population size of colonizing force (*i.e.,* initial introduced population).

[3]Duration of bottleneck event (years).

[4]Admixture coefficient.

[5]Time in the past that an event occurred (years).

Step 1

The first step aimed at identifying which part(s) of the native range have played a role in the introduction of *C. formosanus*. The US introduced range was pooled as a single population (hereafter 'INT'), while the native range and Japan was split into the two regions identified by the PCA analysis – southcentral China (hereafter called 'SC'; Beihai, Changsha, Guilin, Hainan, Hengyang and Nanning) and eastern Asia (hereafter called 'EA'; Fuzhou, Hangzhou, Wenzhou, Hong Kong, Jieyang, Lufeng, Taiwan, Xiamen, Okinawa and mainland Japan). The Mississippi and Xinyu samples were not included in any group as only one sample was available for each location (for this and all subsequent steps). Three scenarios were simulated and tested during this first step. The emergence of the introduced population from a single introduction event followed by a bottleneck out of EA, SC or a combination and admixture or both regions was compared. Both EA and SC were found to contribute to the introduced population and were therefore kept for further analyses. In the LDA plot on the next page, although the observed dataset (represented by the black star) is separate from most of the simulations (colored pluses), it is closest to the simulated datasets for the S1*c* scenario.

Supplementary Table 2: Prior error rates, RF votes and the posterior probability (for the winning scenario) for the three scenarios tested in step 1.

Scenario	Prior error rate, %	RF votes	Posterior probability
S1*a*: Introduction from EA alone	2.95	79	-
S1*b*: Introduction from SC alone	2.85	46	-
**S1*c*: Introduction from EA + SC**	**1.27**	**375**	**0.55**

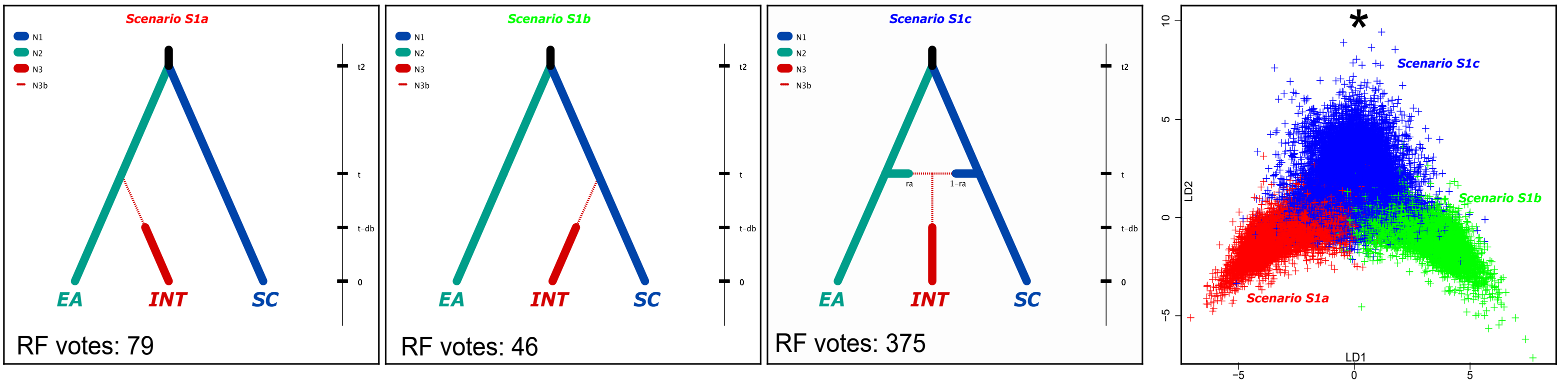

Supplementary Figure 3: **Step 1.** The three scenarios compared in step 1, along with the LDA plot of the simulated (colored crosses) and observed (black star) datasets.

Step 2

The second and third steps aimed at determining whether the Hong Kong region (hereafter called 'HK'; Hong Kong, Jieyang, Lufeng, Taiwan, Xiamen and Okinawa) is the sole source of the introduced population of *C. formosanus.* Therefore, HK was separated from the other eastern Chinese localities (hereafter called sub-eastern Asia '*sub*EA'; Fuzhou, Hangzhou, Wenzhou and mainland Japan). This separation is based on the substructure present within the EA cluster in the PCA, fastSTRUCTURE and fineRADstructure, as well as the clustering of the introduced population out of the HK branch in the maximum likelihood tree. The second step tested for the emergence of the introduced population from admixture between only two of the three native populations (HK+SC *vs.* HK+*sub*EA *vs. sub*EA+SC). The two-population admixture scenario between HK and s*ub*EA was found most probable and will be compared to a three-population admixture scenario in the third step. In the LDA plot on the next page, the observed dataset is closest to the simulated datasets for the S2*c* scenario.

Supplementary Table 3: Prior error rates, RF votes and the posterior probability (for the winning scenario) for the three scenarios tested in step 2.

Scenario	Prior error rate, %	RF votes	Posterior probability
S2*a*: Introduction from *sub*EA + SC	2.86	126	-
S2*b*: Introduction from HK + SC	2.32	89	-
**S2*c*: Introduction from HK+*sub*EA**	**2.36**	**285**	**0.57**

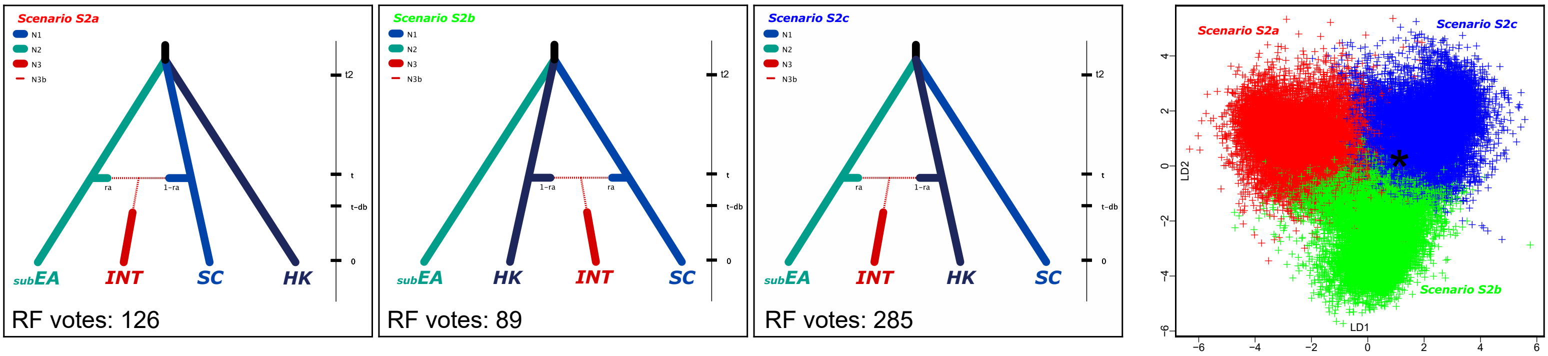

Supplementary Figure 4: **Step 2.** The three scenarios compared in step 2, along with the LDA plot of the simulated (colored crosses) and observed (black star) datasets.

Sub-step 2

Sub-steps 2A and 2B sought to determine whether either of the Japanese populations were driving step 2's result and required separation from the Hong Kong region group (for Okinawa) or sub-eastern Asian group (for mainland Japan). Okinawa and mainland Japan were split between the two eastern Asian regions due to their distinct clustering in fastSTRUCTURE, fineRADstructure and the PCA. Therefore, Okinawa (**Supplementary Table 4**) and mainland Japan (**Supplementary Table 5**) were separated from their groups and compared against their remaining groupmates under the best model from step 2. In both sub-steps, neither Okinawa nor mainland Japan alone explained the invasion history better than their group, justifying their inclusion in the groups defined in step 2 and not their separation into distinct groups. In both LDA plots on the next page, the observed datasets are closest to the simulated datasets for the winning scenarios (Sub2A-*a* and Sub2B-*a*).

Supplementary Table 4: Prior error rates, RF votes and the posterior probability (for the winning scenario) for the two scenarios tested in sub-step 2A (Okinawa separation).

Scenario	Prior error rate, %	RF votes	Posterior probability
**Sub2A-*a*: Introduction from *sub*EA + HK (without Okinawa)**	**3.27**	**346**	**0.58**
Sub2A-*b*: Introduction from *sub*EA + Okinawa	2.99	154	-

Supplementary Table 5: Prior error rates, RF votes and the posterior probability (for the winning scenario) for the two scenarios tested in sub-step 2B (mainland Japan separation).

Scenario	Prior error rate, %	RF votes	Posterior probability
**Sub2B-*a*: Introduction from *sub*EA (without mainland Japan) + HK**	**2.89**	**251**	**0.64**
Sub2B-*b*: Introduction from mainland Japan + HK	3.83	249	-

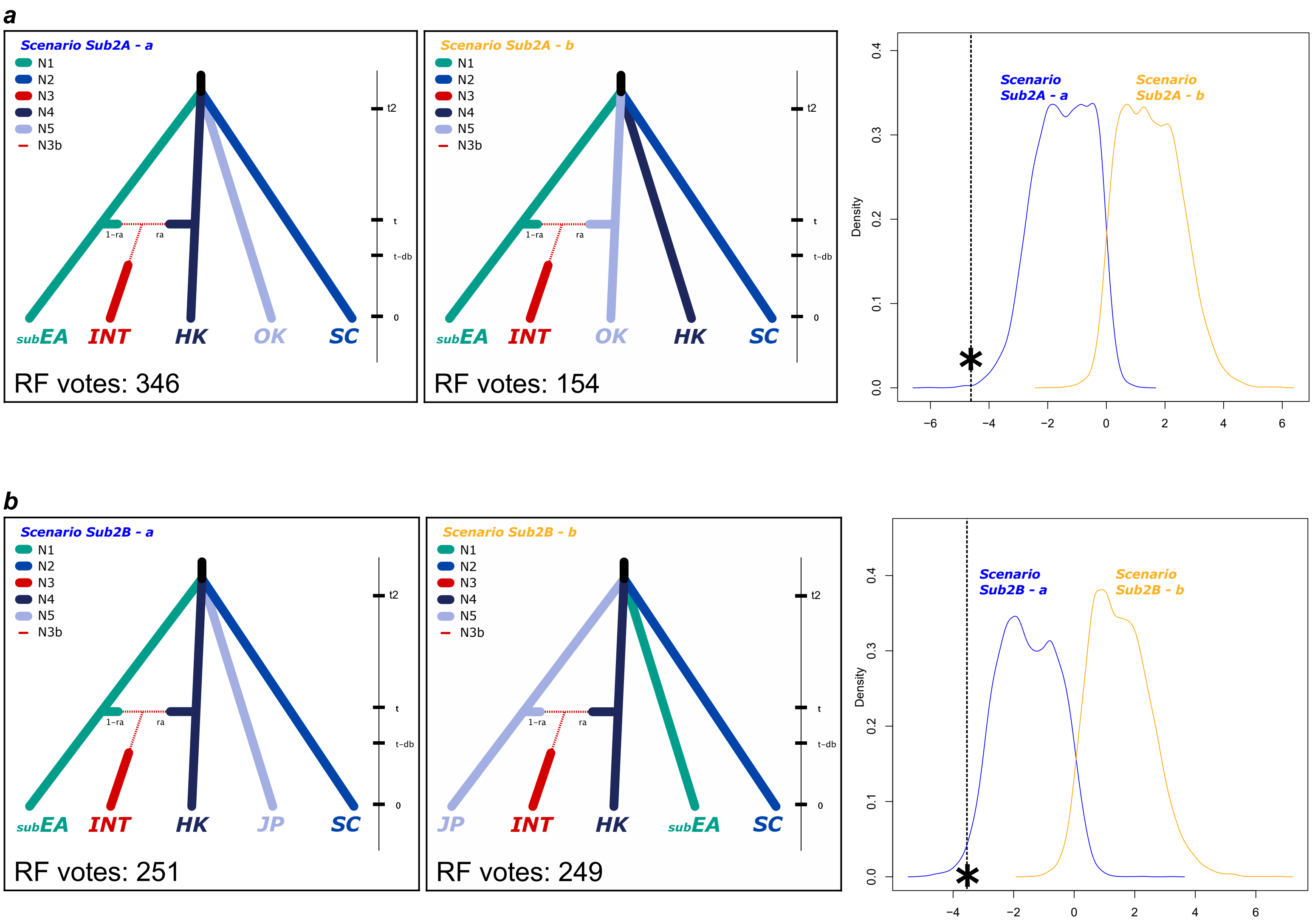

Supplementary Figure 5: **Sub-step 2. a** The two scenarios compared in sub-step 2A, along with the LDA plot of the simulated (blue and gold lines) and observed (black star) datasets. **b** The two scenarios compared in sub-step 2B, along with the LDA plot of the simulated (blue and gold lines) and observed (black star) datasets.

Step 3

The third step then compared the best two-population admixture scenario with scenarios simulating the origin of the introduced population from admixture between all three populations. As DIYABC does not allow for the simultaneous admixture of three populations, we divided the simulations into three scenarios interchanging which two-population admixture comes first [1) (HK+SC)+*sub*EA, 2) (HK+*sub*EA)+SC, 3) (*sub*EA+SC)+HK]. These three similar scenarios limit the number of RF votes each can obtain individually, therefore we calculated the number of RF votes for an overall three-population admixture as the sum of all three scenarios (cumulative RF votes: 469 votes). This step suggested that the introduced range contained elements of all three native clusters (consistent with Step 1), therefore all were retained for further analyses. In the LDA plot on the next page, the observed dataset is closest to the simulated datasets for the three-population admixture scenarios (S3*b*, S3*c* and S3*d*).

Supplementary Table 6: Prior error rates, RF votes and the posterior probability (for the winning scenario) for the four scenarios tested in step 3.

Scenario	Prior error rate, %	RF votes	Posterior probability
S3*a*: Introduction from best two-population admixture HK + *sub*EA	17.82	31	-
**S3*b*: Introduction from *sub*EA + SC first, then HK**	**45.76**	**153**	**-**
**S3*c*: Introduction from HK + *sub*EA first, then SC**	**42.99**	**166**	**0.55**
**S3*d*: Introduction from HK + SC first, then *sub*EA**	**45.06**	**150**	**-**

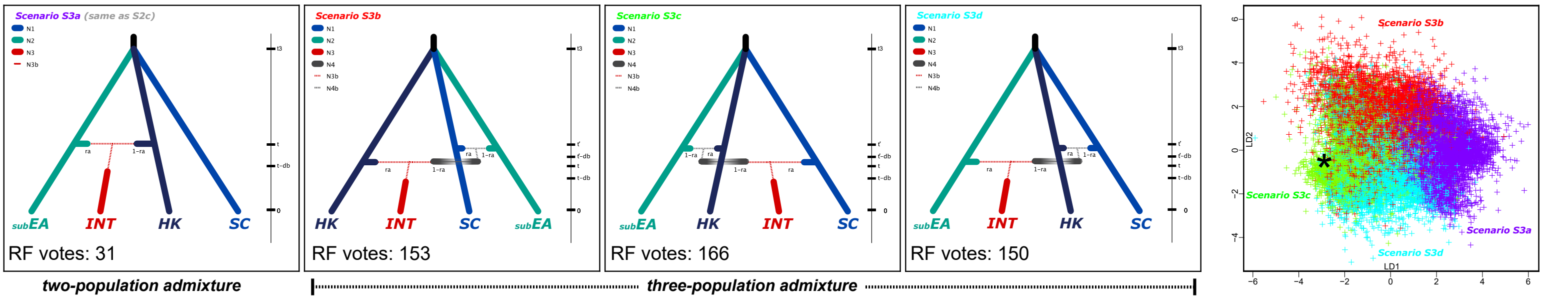

Supplementary Figure 6: **Step 3.** The four scenarios compared in step 3, along with the LDA plot of the simulated (colored crosses) and observed (black star) datasets.

Step 4

The fourth step aimed at identifying whether the US mainland descended from a Hawaiian bridgehead or from a separate invasion out of the native range. This step also tested whether the Hawaii (hereafter called ‘HI’) and the US mainland (hereafter called ‘US’) arose from the same part of Asia (either EA or SC), from a different part, or from admixture between the two (EA+SC). The HI population was therefore separated from the US population, and HK was reintegrated into EA to reduce computational effort. Two scenarios obtained a similarly high number of votes. One scenario (S4c) depicted HI originating from EA, with US originating independently from SC. The second scenario (S4g) depicted US arising from an already introduced, admixed HI population through bridgehead. Therefore, we retained both scenarios for further analyses. In the LDA plot on the next page, the observed dataset is closest to the simulated datasets for scenarios S4*a*, S4*e* and S4*g*.

Supplementary Table 7: Prior error rates, RF votes and the posterior probability (for the winning scenario) for the seven scenarios tested in step 4.

Scenario	Prior error rate, %	RF votes	Posterior probability
S4*a*: US and HI arose independently from EA	8.67	37	-
S4*b*: US and HI arose independently from SC	8.54	69	-
**S4*c*: US arose from SC, HI arose from EA**	**0.3**	**155**	**0.71**
S4*d*: US arose from EA, HI arose from SC	0.13	25	-
S4*e*: US arose from HI bridgehead, HI arose from EA	6.25	18	-
S4*f*: US arose from HI bridgehead, HI arose from SC	5.32	60	-
**S4*g*: US arose from HI bridgehead, HI arose from admixture between EA and SC**	**1.23**	**136**	**-**

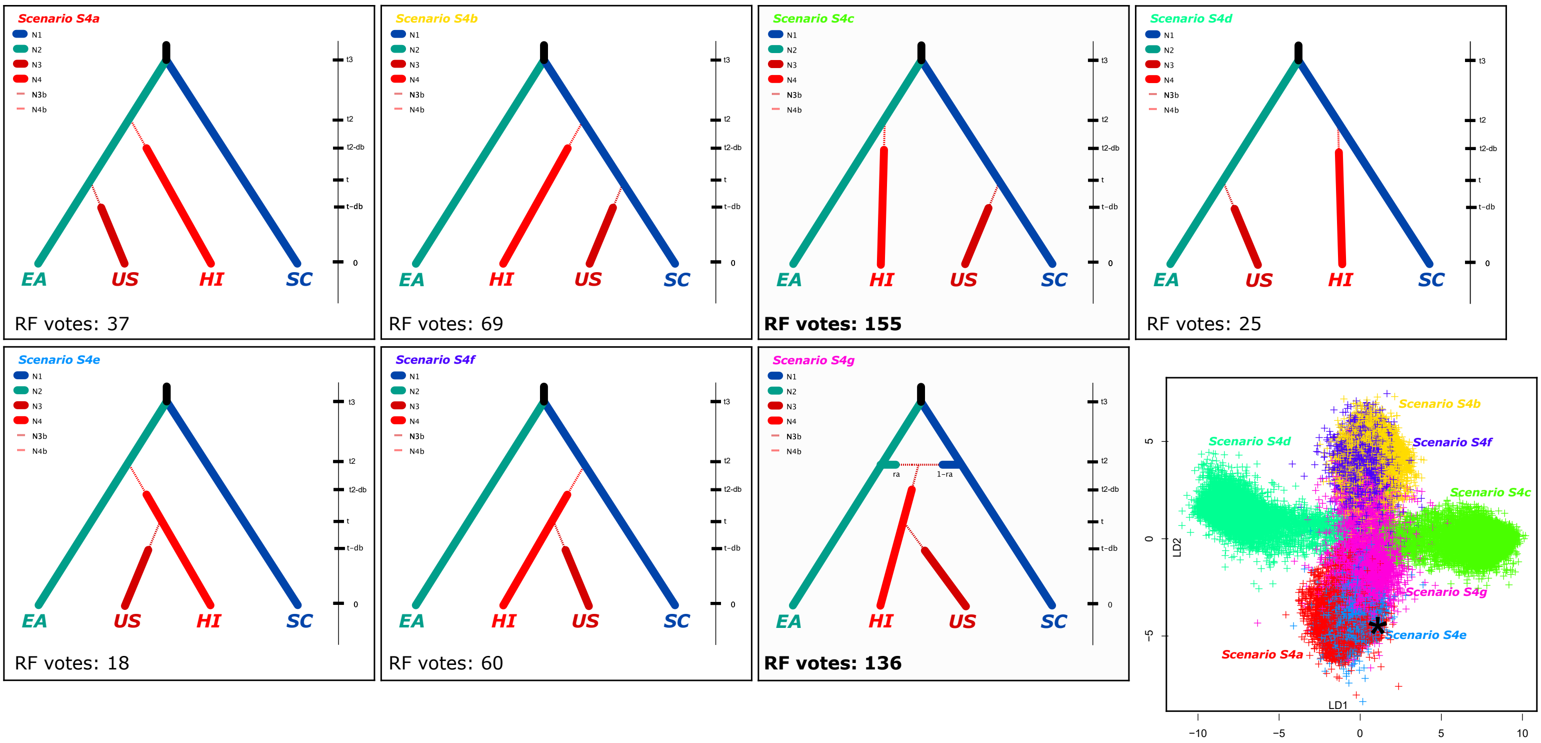

***Supplementary Figure 7:* Step 4.** The seven scenarios compared in step 4, along with the LDA plot of the simulated (colored crosses) and observed (black star) datasets.

Sub-step 4

Sub-step 4 sought to confirm a Hawaiian bridgehead to the US mainland. Two scenarios in step 4 obtained a similar number of votes, with the difference between the two most likely scenarios seemingly driven by the US mainland. Therefore, US was split into Louisiana/Texas (hereafter called ‘LA+TX’) and Florida (hereafter called ‘FL’). This separation is justified based on the distinct cluster found among some Florida samples at the best value of K (15) in fastSTRUCTURE and in the DAPC. Additionally, as HI and LA+TX clustered the same at the best K and HI appears more related to EA samples (step 4, scenario *c*), both groups were descended from EA in all scenarios below, with FL as a constant descended from SC (reflecting the aforementioned discrepancy). The third scenario (Sub4*c*) depicting a HI bridgehead to LA+TX was found most likely, confirming at least one bridgehead from HI to the US mainland has occurred. In the LDA plot on the next page, the observed dataset is closest to the simulated datasets for scenario Sub4*c*.

Supplementary Table 8: Prior error rates, RF votes and the posterior probability (for the winning scenario) for the four scenarios tested in sub-step 4.

Scenario	Prior error rate, %	RF votes	Posterior probability
Sub4*a*: HI and LA+TX arose independently and simultaneously from EA, FL arose from SC	11.37	52	-
Sub4*b*: HI and LA+TX arose independently from EA (HI first), FL arose from SC	14.59	117	-
**Sub4*c*: HI arose from EA, LA+TX arose from HI bridgehead, FL arose from SC**	**2.61**	**194**	**0.32**
Sub4*d*: HI and LA+TX arose independently from EA (LA+TX first), FL arose from SC	15.29	137	-

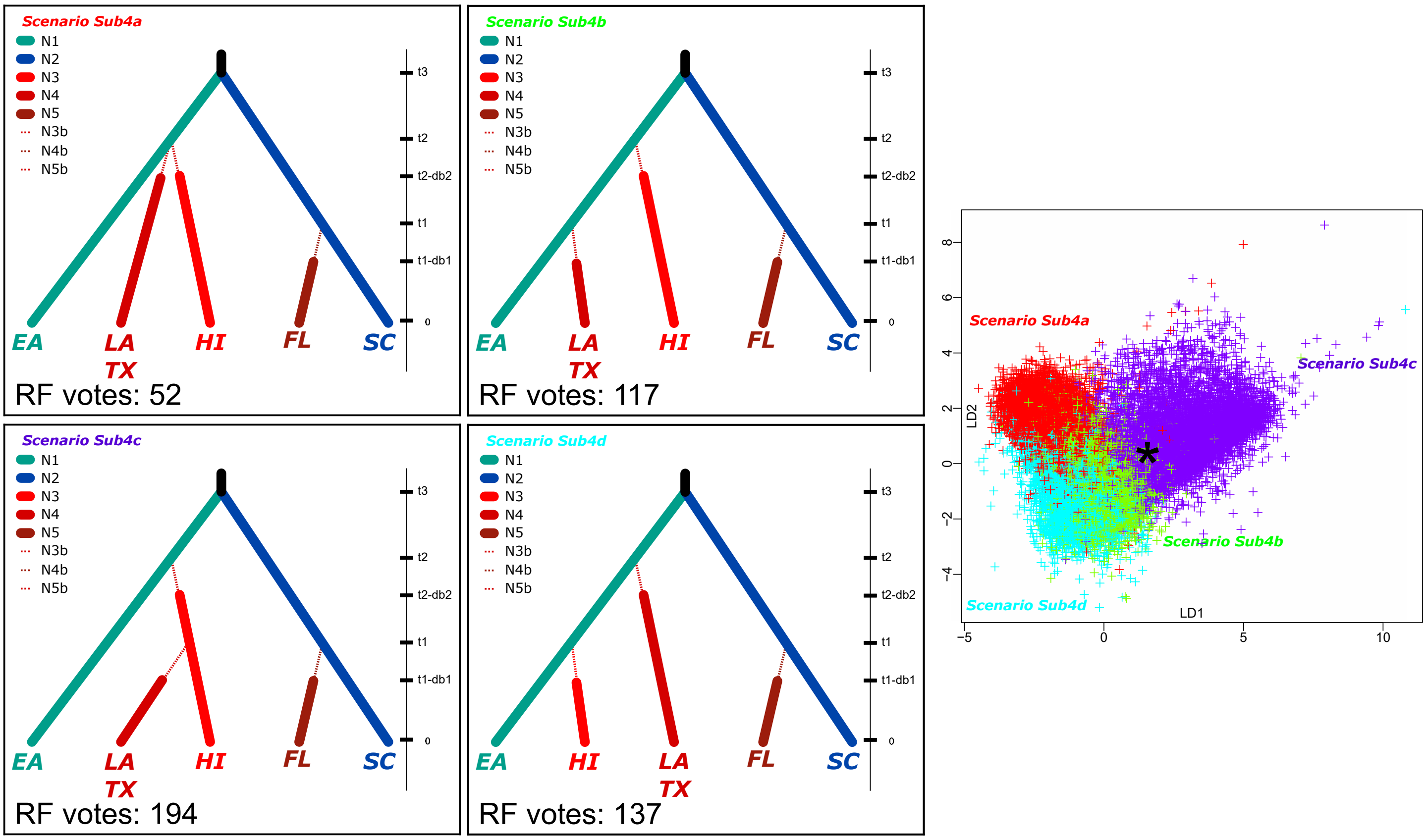

Supplementary Figure 8: **Sub-step 4.** The four scenarios compared in sub-step 4, along with the LDA plot of the simulated (colored crosses) and observed (black star) datasets.

Step 5

Similar to step 3 above, the fifth step further investigated whether HK and *sub*EA played distinct roles in the introduction of *C. formosanus* to HI. This step used the most likely scenario obtained from sub-step 4 (HI bridgehead to LA+TX) and again split the overall EA region into HK and *sub*EA (and kept FL as a constant descended from SC). This step confirmed that both sub-regions of eastern Asia played a role in the invasion of HI. In the LDA plot on the next page, the observed dataset is closest to the simulated datasets for scenario S5*c*.

Supplementary Table 9: Prior error rates, RF votes and the posterior probability (for the winning scenario) for the three scenarios tested in step 5.

Scenario	Prior error rate, %	RF votes	Posterior probability
S5*a*: HI arose from *sub*EA, LA+TX arose from HI bridgehead, FL arose from SC	7.44	146	-
S5*b*: HI arose from HK, LA+TX from HI bridgehead, FL arose from SC	6.83	63	-
**S5*c*: HI arose from admixture between *sub*EA and HK, LA+TX arose from HI bridgehead, FL arose from SC**	**3.62**	**291**	**0.38**

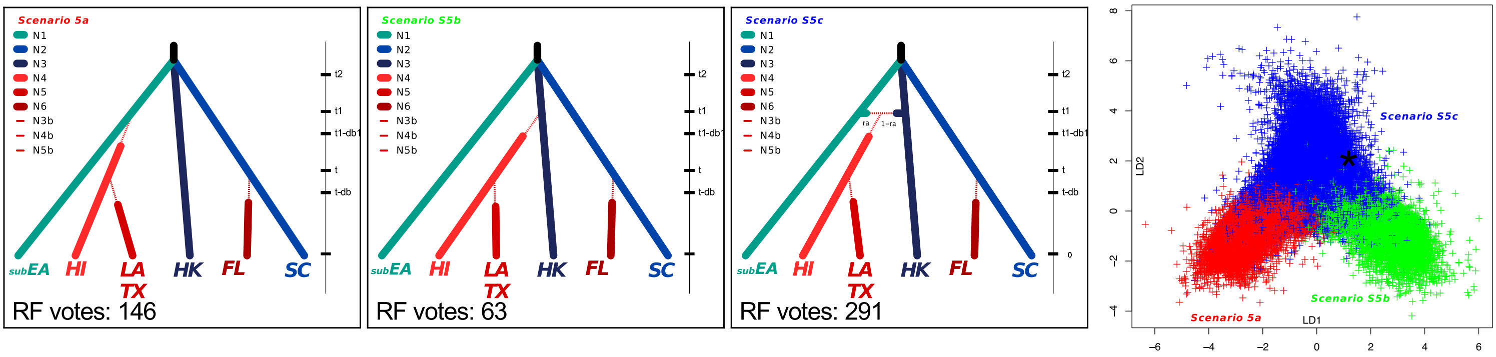

Supplementary Figure 9: **Step 5.** The three scenarios compared in step 5, along with the LDA plot of the simulated (colored crosses) and observed (black star) datasets.

Step 6

This sixth step investigated whether the FL introduction event is connected with the invasion of HI and LA+TX, or separate. Therefore, we compared the best scenario from step 5 (S5*c*) with bridgehead introduction events from either HI or LA+TX that admixed with a separate introduction event from SC. The SC influence in Florida (as opposed to LA+TX) is supported by the distinct clustering of some Florida samples at best K in fastSTRUCTURE, as well as results from step 4 above. The sixth step found it most likely that FL is linked with the invasion of HI and LA+TX, with FL resulting from admixture between LA+TX and SC. In the LDA plot on the next page, the observed dataset is near the midpoint of all three scenarios.

Supplementary Table 10: Prior error rates, RF votes and the posterior probability (for the winning scenario) for the three scenarios tested in step 6.

Scenario	Prior error rate, %	RF votes	Posterior probability
S6*a*: HI arose from admixture between *sub*EA and HK, LA+TX arose from HI bridgehead, FL arose from SC	11.12	125	-
S6*b*: HI arose from admixture between *sub*EA and HK, LA+TX arose from HI bridgehead, FL arose from admixture between HI and SC	19.78	155	-
**S6*c*: HI arose from admixture between *sub*EA and HK, LA+TX arose from HI bridgehead, FL arose from admixture between LA+TX and SC**	**13.72**	**220**	**0.44**

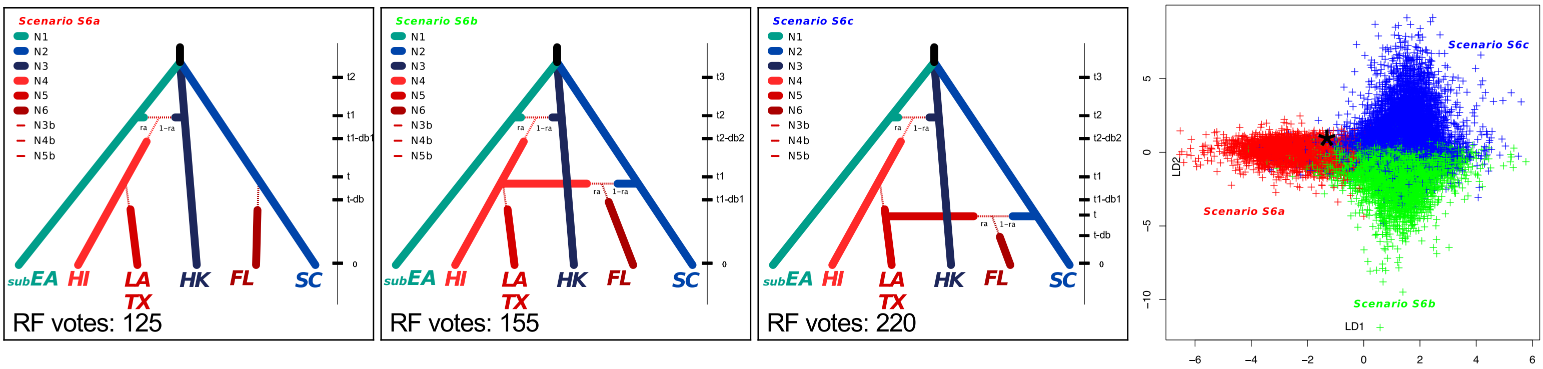

Supplementary Figure 10: **Step 6.** The three scenarios compared in step 6, along with the LDA plot of the simulated (colored crosses) and observed (black star) datasets.

Supplementary Table 11: Parameter estimates for the final invasion model (S6*c*).

Population	Parameter	Estimate
-	t4	3700
Hong Kong region	N1	5868
Southcentral China	N2	7229
Sub-eastern Asia	N3	5114
Hawaii	N4	4962
	t3	138
	N4b	54
	db3	18
	ra2	0.52
Louisiana+Texas	N5	4671
	t2	98
	N5b	52
	db2	16
Florida	N6	4976
	t1	87
	N6b	52
	db1	18
	ra1	0.49

DAPC analysis

Number of genetic clusters

We utilized the Bayesian information criterion (BIC) to select the most likely number of genetic clusters. After 15 clusters, the BIC begins to dramatically increase, supporting our selection of 15 clusters.

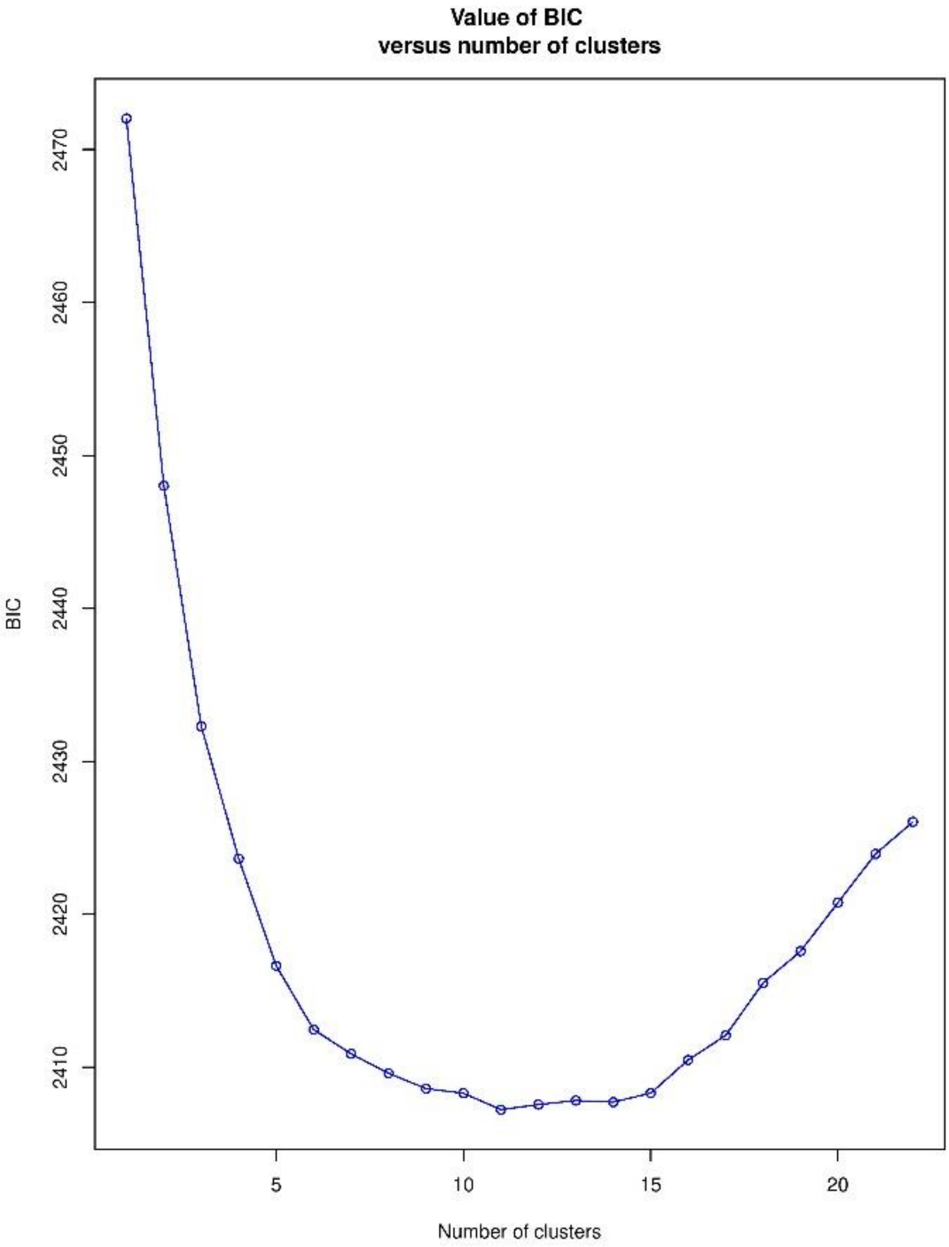

Supplementary Figure 11: Genetic clusters. The BIC value for each cluster. Note that the BIC begins to dramatically increase after 15.

Principal component optimization

We utilized the a-score to optimize the number of principal components (PCs) to include in our DAPC – 50 PCs maximized the a-score and were therefore selected.

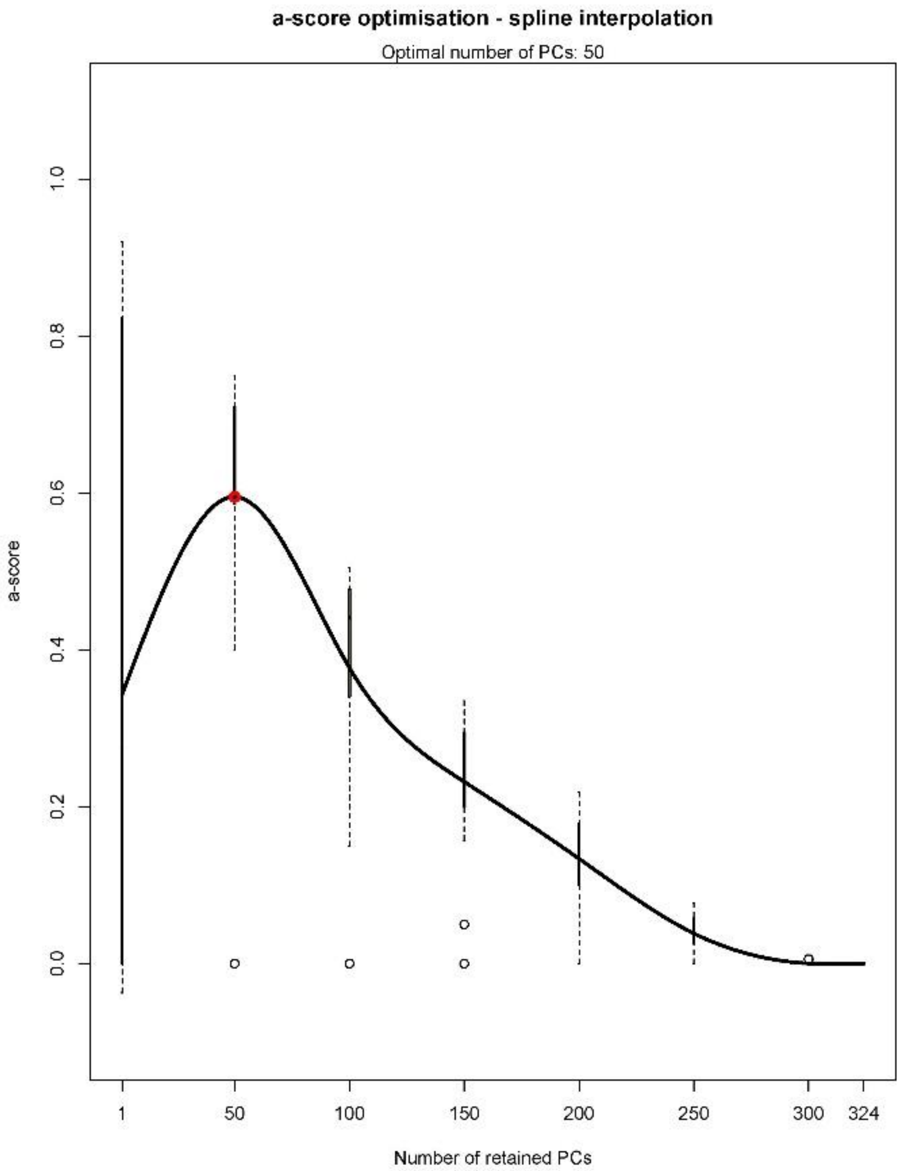

Supplementary Figure 12: Principal component optimization. The red dot represents the number of retained PCs that maximize the a-score.

Supplementary Table 12: The number of *C. formosanus* workers sampled from each location.

Locality	N
United States	
Hawaii	14
Texas	4
Louisiana	16
Mississippi	1
Florida	46
Japan	
Okinawa	18
Mainland	20
China	
Nanning	20
Beihai	20
Hainan	14
Guilin	20
Hengyang	20
Changsha	3
Xinyu	1
Hong Kong	20
Lufeng	7
Jieyang	15
Xiamen	20
Fuzhou	20
Wenzhou	20
Hangzhou	20
Taiwan	20

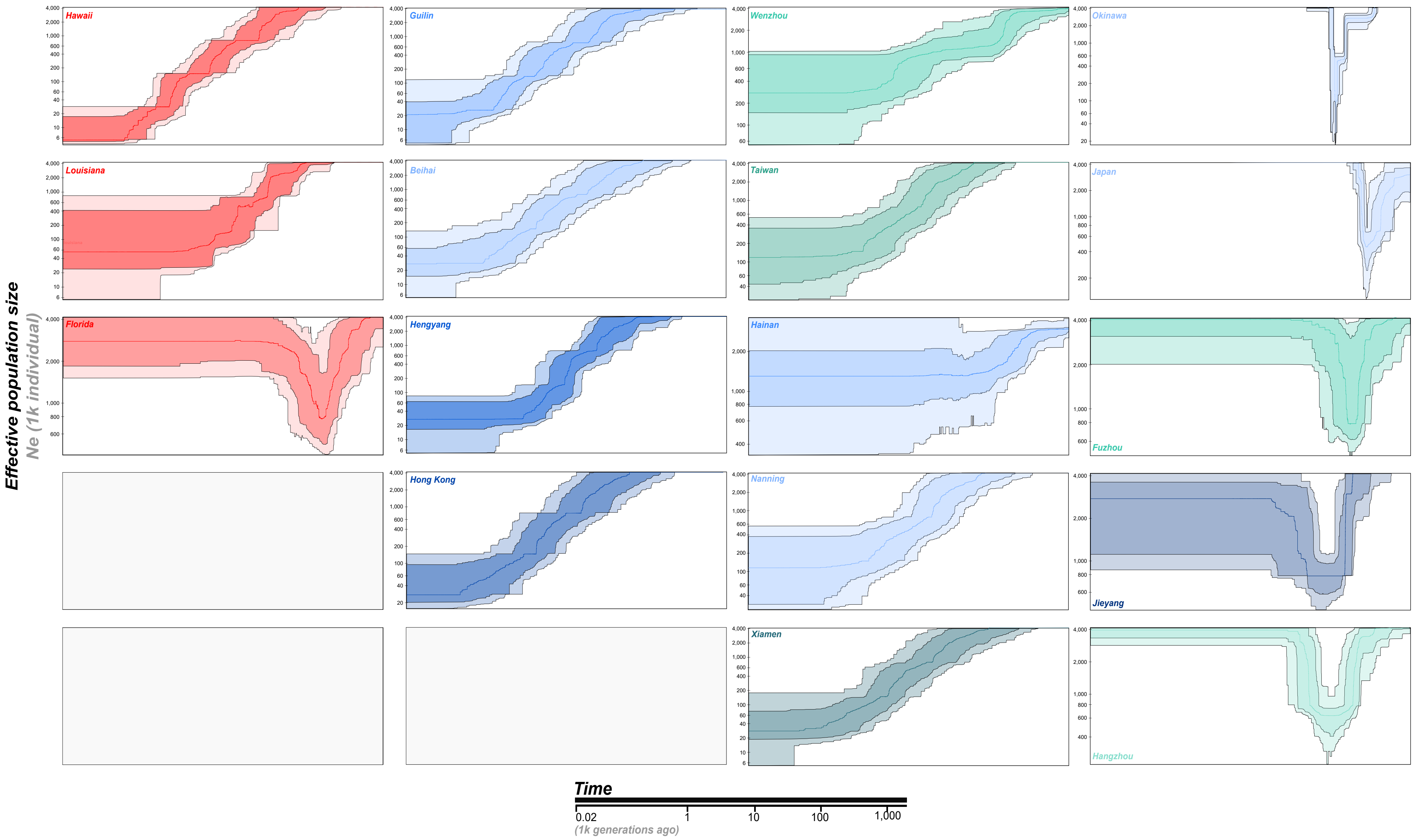

Supplementary Figure 13: **Demographic histories.** Estimation of the variation of the effective population size through time for all invasive and native localities using Stairway Plot 2. Light and dark shaded areas for each plot correspond to the 95% and 75% confidence intervals for the effective population size, respectively. The dark line within the confidence intervals in each plot represents the estimate for the median effective population size.
