# Supplementary material for: Bridgehead effect and multiple introductions shape the global invasion history of a termite
Source: Commun Biol. 2021 Feb 12;4:196. doi: 10.1038/s42003-021-01725-x (PMC7881189; doi:10.1038/s42003-021-01725-x)
Supplement: Supplementary file 3 — Reporting Summary [file 42003_2021_1725_MOESM3_ESM.pdf]

Corresponding author(s): Alexander J. Blumenfeld

Last updated by author(s): Dec 10, 2020

Reporting Summary

Nature Research wishes to improve the reproducibility of the work that we publish. This form provides structure for consistency and transparency in reporting. For further information on Nature Research policies, see our Editorial Policies and the Editorial Policy Checklist.

Statistics

For all statistical analyses, confirm that the following items are present in the figure legend, table legend, main text, or Methods section.

n/a	Confirmed	
☐	☒	The exact sample size (*n*) for each experimental group/condition, given as a discrete number and unit of measurement
☐	☒	A statement on whether measurements were taken from distinct samples or whether the same sample was measured repeatedly
☒	☐	The statistical test(s) used AND whether they are one- or two-sided<br>*Only common tests should be described solely by name; describe more complex techniques in the Methods section.*
☒	☐	A description of all covariates tested
☒	☐	A description of any assumptions or corrections, such as tests of normality and adjustment for multiple comparisons
☒	☐	A full description of the statistical parameters including central tendency (e.g. means) or other basic estimates (e.g. regression coefficient) AND variation (e.g. standard deviation) or associated estimates of uncertainty (e.g. confidence intervals)
☒	☐	For null hypothesis testing, the test statistic (e.g. *F*, *t*, *r*) with confidence intervals, effect sizes, degrees of freedom and *P* value noted<br>*Give P values as exact values whenever suitable.*
☐	☒	For Bayesian analysis, information on the choice of priors and Markov chain Monte Carlo settings
☒	☐	For hierarchical and complex designs, identification of the appropriate level for tests and full reporting of outcomes
☒	☐	Estimates of effect sizes (e.g. Cohen's *d*, Pearson's *r*), indicating how they were calculated

Our web collection on statistics for biologists contains articles on many of the points above.

Software and code

Policy information about availability of computer code

Data collection	No software used for data collection.
Data analysis	FastQC v0.11.849, Stacks v.2.41, PGDSpider v.2.1.1.5, VCFtools v.0.1.15, fastSTRUCTURE v1.040, Structure_threader, Distruct v2.3, fineRADstructure v0.3.2, RAxML v8.2.12, Phrynomics (https://github.com/bbanbury/phrynomics/), DIYABC v2.0, ABC RF v.1.8.1, R v.3.6.2, Stairway Plot 2

For manuscripts utilizing custom algorithms or software that are central to the research but not yet described in published literature, software must be made available to editors and reviewers. We strongly encourage code deposition in a community repository (e.g. GitHub). See the Nature Research guidelines for submitting code & software for further information.

Data

Policy information about availability of data

All manuscripts must include a data availability statement. This statement should provide the following information, where applicable:

- Accession codes, unique identifiers, or web links for publicly available datasets
- A list of figures that have associated raw data
- A description of any restrictions on data availability

The derived data reported in this study have been deposited in the Open Science Framework database, https://osf.io (DOI 10.17605/OSF.IO/QSBD5). Sequence data has been deposited in NCBI (BioProject accession number PRJNA666619).

Field-specific reporting

Please select the one below that is the best fit for your research. If you are not sure, read the appropriate sections before making your selection.

☐ Life sciences ☐ Behavioural & social sciences ☒ Ecological, evolutionary & environmental sciences

For a reference copy of the document with all sections, see nature.com/documents/nr-reporting-summary-flat.pdf

Ecological, evolutionary & environmental sciences study design

All studies must disclose on these points even when the disclosure is negative.

Study description	Elucidating the invasion history of Coptotermes formosanus from eastern Asia to the USA.
Research sample	In the native range, termite colonies were sampled across thirteen localities in mainland China (southcentral China – Beihai, Changsha, Guilin, Hainan, Hengyang, Nanning and Xinyu; eastern China – Fuzhou, Hangzhou, Jieyang, Lufeng, Wenzhou and Xiamen), Hong Kong and Taiwan. In the introduced range, colonies were sampled in mainland Japan and Okinawa, as well as in Hawaii, Texas, Louisiana, Mississippi and Florida.
Sampling strategy	Sampling locations were chosen to obtain a representative sample from both the native and invasive range of the species.
Data collection	Samples collected mainly by Edward Vargo and Claudia Husseneder, with the help of some regional collaborators.
Timing and spatial scale	Samples were obtained from 2005 to 2016.
Data exclusions	Individuals were excluded if sequencing was of poor quality (i.e., high missing data %) - 34 individuals ended up being excluded from the final analysis (and 325 individuals utilized).
Reproducibility	No experiments.
Randomization	No randomization, samples were assigned geographically.
Blinding	Blinding not used/not relevant.

Did the study involve field work? ☒ Yes ☐ No

Field work, collection and transport

Field conditions	Mostly urban areas (e.g., wooden buildings, urban trees).
Location	Various cities/regions (see above) in China, Japan & the USA.
Access & import/export	Collections were made in China and Japan in accordance with laws of the time period (2005-2010), and samples imported into the US in compliance with US regulations.
Disturbance	No disturbance.

Reporting for specific materials, systems and methods

We require information from authors about some types of materials, experimental systems and methods used in many studies. Here, indicate whether each material, system or method listed is relevant to your study. If you are not sure if a list item applies to your research, read the appropriate section before selecting a response.

Materials & experimental systems

n/a	Involved in the study	
☒	☐	Antibodies
☒	☐	Eukaryotic cell lines
☒	☐	Palaeontology and archaeology
☐	☒	Animals and other organisms
☒	☐	Human research participants
☒	☐	Clinical data
☒	☐	Dual use research of concern

Methods

n/a	Involved in the study	
☒	☐	ChIP-seq
☒	☐	Flow cytometry
☒	☐	MRI-based neuroimaging

Animals and other organisms

Policy information about studies involving animals; ARRIVE guidelines recommended for reporting animal research

Laboratory animals	No laboratory animals.
Wild animals	Termites were collected from wild colonies and placed in tubes of ethanol for killing and transport.
Field-collected samples	Termites were stored in ethanol until DNA extraction and library preparation.
Ethics oversight	No guidance required because termites are insects.

Note that full information on the approval of the study protocol must also be provided in the manuscript.
